# The CTCF/LncRNA‐PACERR complex recruits E1A binding protein p300 to induce pro‐tumour macrophages in pancreatic ductal adenocarcinoma via directly regulating PTGS2 expression

**DOI:** 10.1002/ctm2.654

**Published:** 2022-02-20

**Authors:** Yihao Liu, Xuelong Wang, Youwei Zhu, Yizhi Cao, Liwen Wang, Fanlu Li, Yu Zhang, Ying Li, Zhiqiang Zhang, Jiaxin Luo, Xiaxing Deng, Chenghong Peng, Gang Wei, Hao Chen, Baiyong Shen

**Affiliations:** ^1^ Department of General Surgery Pancreatic Disease Center Ruijin Hospital Shanghai Jiao Tong University School of Medicine Shanghai China; ^2^ CAS Key Laboratory of Computational Biology Shanghai Institute of Nutrition and Health, University of Chinese Academy of Sciences Chinese Academy of Sciences Shanghai China; ^3^ Research Institute of Pancreatic Diseases Shanghai Jiao Tong University School of Medicine Shanghai China; ^4^ State Key Laboratory of Oncogenes and Related Genes, Institute of Translational Medicine Shanghai Jiao Tong University Shanghai China; ^5^ Institute of Translational Medicine Shanghai Jiaotong University Shanghai China

**Keywords:** CTCF, E1A binding protein p300, Epigenetic mechanisms, LncRNA‐PACERR, pancreatic ductal adenocarcinoma, prostaglandin‐endoperoxide synthase 2, tumour‐associated macrophages

## Abstract

**Background:**

Tumour‐associated macrophages (TAMs) play an important role in promoting the progression of pancreatic ductal adenocarcinoma (PDAC). Here, we aimed to study the epigenetic mechanisms in regulating pro‐tumour M2‐polarised TAMs in the PDAC tumour microenvironment.

**Methods:**

This study was conducted based on ex vivo TAMs isolated from PDAC tissues and in vitro THP1‐derived TAM model. RNA‐sequencing (RNA‐seq), assay for transposase‐accessible chromatin with sequencing and chromatin immunoprecipitation sequencing were performed to investigate gene expression, chromatin accessibility, transcription factor binding sites and histone modifications. Gene knockdown in THP1‐derived TAMs was performed with lentivirus, and the impact of THP1‐derived TAMs on invasion and metastasis ability of PDAC cells were investigated with in vitro and in vivo functional assays. RNA‐chromatin interaction was analysed by chromatin isolation through RNA purification with sequencing. RNA‐protein interaction was studied by RNA immunoprecipitation and RNA pull‐down.

**Results:**

Our data showed that the transcription factor CTCF (CCCTC‐binding factor) was highly expressed in TAMs and predicted to be significantly enriched in hyper‐accessible chromatin regions when compared to monocytes. High infiltration of CTCF^+^ TAMs was significantly associated with poor prognosis in PDAC patients. Knockdown of CTCF in THP1‐derived TAMs led to the down‐regulation of specific markers for M2‐polarised TAMs, including CD206 and CD163. When THP1‐derived TAMs with CTCF knockdown, they showed a decreased ability of invasion and metastasis. Further integrative analysis of multi‐omics data revealed that prostaglandin‐endoperoxide synthase 2 (PTGS2) and PTGS2 antisense NF‐κB1 complex‐mediated expression regulator RNA (PACERR) were critical downstream targets of CTCF and positively correlated with each other, which are closely situated on a chromosome. Knockdown of PACERR exhibited a similar phenotype as observed in CTCF knockdown THP1‐derived TAMs. Moreover, PACERR could directly bind to CTCF and recruit histone acetyltransferase E1A binding protein p300 to the promoter regions of PACERR and PTGS2, thereby enhancing histone acetylation and gene transcription, promoting the M2 polarization of TAMs in PDAC.

**Conclusions:**

Our study demonstrated a novel epigenetic regulation mechanism of promoting pro‐tumour M2‐polarised TAMs in the PDAC tumour microenvironment.

## Graphical abstract

In this study, based on integrative multi‐omics analysis and solid experimental validation, we revealed a novel epigenetic mechanism of regulating PTGS2 (COX‐2) expression in TAMs, thereby inducing pro‐tumor M2‐polarized TAMs in PDAC tumor microenvironment. Considering the significant off‐target side effects of currently available agents for PTGS2 (COX‐2) blockade, our data provides rationales for designing novel therapeutic approaches interfering pro‐tumor functions of TAMs in PDAC.

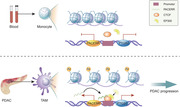

**List of abbreviations**
ATAC‐seq: assay for transposase‐accessible chromatin with sequencing
ChIP: chromatin immunoprecipitation
ChIRP‐seq: chromatin isolation through RNA purification with sequencing
CTCF: CCCTC‐binding factor
DMEM: Dulbecco's Modified Eagle Medium
ELISA: enzyme‐linked immunosorbent assay
EMT: epithelial‐mesenchymal transition
EP300: E1A binding protein p300
FBS: Fetal Bovine Serum
HAT: histone acetyltransferase
LncRNA: long noncoding RNA
MACS: magnetic‐activated cell sorting
PACERR: PTGS2 antisense NFKB1 complex‐mediated expression regulator RNA
PDAC: pancreatic ductal adenocarcinoma
PE: phycoerythrin
PFA: paraformaldehyde
PMA: phorbol 12‐myristate 13‐acetate
PTGS2: prostaglandin‐endoperoxide synthase 2
RIP: RNA immunoprecipitation
RNA‐FISH: RNA‐fluorescence in situ hybridisation
SJTU: Shanghai Jiao Tong University
TAMs: tumour‐associated macrophages
TCGA: The Cancer Genome Atlas
TFs: transcription factors
TMAs: Tissue microarrays
TSS: transcription start site
WT: wild‐type

## 1 BACKGROUND

Pancreatic cancer, comprising mostly pancreatic ductal adenocarcinoma (PDAC), is one of the most aggressive and lethal malignancies, with insidious onset, rapid progression, and extremely poor prognosis of a dismal 5‐year survival rate of 8%.^1^, ^2^ The current standard of care for PDAC patients include surgery and chemotherapy, which have only provided marginal survival benefits.^3^ Unfortunately, despite the ground‐breaking achievements of immunotherapy and target therapy, these newly‐emerged treatment regimens have also failed to yield effective responses in PDAC.^4^, ^5^ Therefore, it is urgently required to develop advanced therapeutic approaches for PDAC patients.

The poor prognosis of PDAC is related to an extensive fibroinflammatory tumour microenvironment, with abundant infiltration of cancer‐associated fibroblasts and immune cells.^6^, ^7^ Heavy desmoplastic reaction not only results in increased interstitial pressure, restricting the delivery of chemotherapeutic agents,^8^, ^9^ but also inhibits anti‐tumour immunosurveillance by sequestering CD8^+^ T cells to abrogate their contact with tumour cells.^10^ In addition, the immune filtrates in the tumour microenvironment are hijacked by malignant cells to provide an immunosuppressive microenvironment supporting tumour growth.^5^, ^11^, ^12^

Tumour‐associated macrophages (TAMs) account for a substantial fraction among various types of immune filtrates in the PDAC tumour microenvironment.^13^, ^14^ TAMs are a highly dynamic population, with the ability to acquire distinct phenotypes and functions in response to environmental stimuli.^15^, ^16^ The vast majority of TAMs in the tumour milieu are polarised towards tumour‐promoting M2 phenotype, which facilitates tumour growth, angiogenesis, immune escape, and metastasis.^12^, ^13^ Higher infiltration of TAMs is associated with a worse prognosis in PDAC.^14^, ^17^ Therefore, TAMs represent an attractive therapeutic target for PDAC treatment.

Different from malignant cells, TAMs show distinct phenotypes in the tumour microenvironment, while rarely presenting with somatic mutations. Accordingly, the regulation of expression profiles of TAMs mostly occurs at the level of epigenetic modification.^18^ The major molecular mechanisms of epigenetic regulation include DNA methylation, histone modification, chromatin remodelling and non‐coding RNA regulation, which work in a coordinated manner so as to precisely regulate gene expression.^19^, ^20^ Previous research on this area has provided evidence that the regulation of M1/M2 polarization of TAMs is associated with histone modification, which causes remodelling of the chromatin structure, influencing the binding of transcriptional machinery with target genes.^15^, ^18^ A better understanding of the complex molecular mechanisms underlying the epigenetic aberrations of TAMs could provide a basis for designing approaches to reverse its pro‐tumour function and boost anti‐tumour immune responses.

In this study, based on epigenomic analysis of TAMs from PDAC tissues, we revealed the activation of CCCTC‐binding factor (CTCF), an important epigenetic regulator, in TAMs. In vitro and in vivo experiments demonstrated that CTCF promotes the M2 polarization and pro‐tumour functions of TAMs. Further mechanistic studies uncovered that CTCF‐transcribed long noncoding RNA (LncRNA) prostaglandin‐endoperoxide synthase 2 (PTGS2) antisense NF‐κB1 complex‐mediated expression regulator RNA (PACERR) participates in *cis*‐regulation of the expression of PTGS2 (also known as COX‐2), a key driver of pro‐tumour TAMs.

## 2 METHODS AND MATERIALS

### 2.1 Clinical samples

Fresh PDAC tissues, PDAC corresponding non‐tumour tissues and peripheral blood were obtained from the patients (in the year 2019–2020) in Ruijin Hospital of Shanghai Jiao Tong University (SJTU) School of Medicine. The PDAC samples were taken within 10 min after tumour or non‐tumour tissues excision and all cells were labelled with CD206‐phycoerythrin (PE) (BD Pharmingen, 555954) and CD11b‐PE (BD Pharmingen, 557321) monoclonal antibodies, and then TAMs and normal tissue‐resident‐macrophages were isolated by anti‐PE microbeads and magnetic‐activated cell sorting (MACS) column, according to manufacturer's protocol. Blood samples were taken within 10 min after drawing blood, and the monocytes in the experiments were immediately separated by CD14 microbeads (Miltenyi, 130‐050‐201). This study was approved by the Research Ethics Committee of the hospital. Informed consent forms were obtained from all patients.

### 2.2 Cell culture and reagents

PANC‐1, PATU‐8988, Pan02, THP‐1 and HEK‐293T cells were gotten from the Institute of Biotechnology, Chinese Academy of Sciences, and were authenticated at SJTU Analysis Core using DNA analysis. Dulbecco's Modified Eagle Medium (DMEM) supplemented with 10% Fetal Bovine Serum (FBS) and antibiotics were used to culture PanC‐1, PATU‐8988, Pan02 and HEK 293T cells. RPMI‐1640 supplemented with 10% FBS and antibiotics were used to culture THP‐1 cell lines. All cell lines were cultured in 37°C constant temperature incubators under 5% carbon dioxide. THP‐1 were cultured in RPMI‐1640 containing 100 ng/ml phorbol 12‐myristate 13‐acetate (PMA) for 48 h to convert them into macrophages.

### 2.3 The isolation and differentiation of bone marrow‐derived macrophages

It was as previously described to isolate bone marrow‐derived macrophages (BMDMs).^21^ The tibia of 6 weeks old C57BL/6 mice were used to flush out BMDMs. Immediately, those cells were cultured in DMEM with 10% of FBS and 25 ng/ml M‐CSFs for 144 h to induce differentiation to macrophages.

### 2.4 Plasmids and stable cell lines

CTCF, PACERR, E1A binding protein p300 (EP300) knockdown plasmids and the NC‐Flag, CTCF‐Flag, CTCF‐Mut‐Flag overexpressed plasmids were gotten from Gene‐Chem, and the sequences targeting related genes were as shown in Table S1.

### 2.5 The LV3‐pGLV‐GFP‐puromycin vectors were used for CTCF, PACERR and EP300 knockdown assays

To produce lentivirus, 293T cells were cultured in a 10‐cm plate without FBS overnight. Note that, 4 μg of target plasmid, 2 μg of PSPAX2 plasmid, 2 μg of PDM2G plasmid and 20 μl of Lipofectamine 2000 (Thermo Fisher Scientific) (μg DNA: μg Lipofectamine 2000 = 1: 2.5) were added to the 10‐cm plate. After 6 h, fresh mediums with serum and antibiotics were added to the 10‐cm plate. After 48 h, lentivirus was collected and stored at –80°C. For transducing lentivirus, THP‐1 cells (untreated with PMA) were cultured in a 25 ml culture flask, and 5 ml of lentivirus suspension (MOI: 100) with polybrene (5 μg/ml; Gene‐Chem) was added into the flask. After 2 days, 5 μg/ml puromycin (Sangon Biotech) was added into RPMI‐1640 to screen for stable cell lines.

### 2.6 Small interfering RNA transfection

CTCF and negative control small interfering RNAs (siRNAs) were synthesized by Gene Chem. There were targeted sequences in Table S1. Lipofectamine 2000 (Invitrogen, USA) were used to transfect BMDMs immediately after being isolated based on the instructions of the manufacturer. Note that, 2 × 10^5^ BMDMs were transfected with 120 pmol siRNA (siCTCF/siNC) in Opti‐MEM with Lipofectamine 2000 in a 6‐well‐plate. Radioimmunoprecipitation assay lysis buffer was used to lyse the BMDMs for Western blotting after 48 h.

### 2.7 Transwell invasion and migration assay

Note that, 5 × 10^5^ PANC‐1/PATU‐8988 wild‐type (WT) cells were seeded to the upper chamber in 200 μl DMEM without serum, 3× 10^5^ THP‐1 cells were seeded to the lower chamber (Corning Life Sciences, Corning, NY, USA) in 400 μl RPMI‐1640 with 20% FBS, and the chambers were put into 37°C constant temperature incubators for 1 day for migration assay. For the transwell invasion assay, the upper chamber was coated with 1.25 mg/ml of Matrigel (50 μl/well) (BD, USA) and cultured for 2 days. Afterwards, the migrated or invaded cells were stained with crystal violet and counted in three random fields.

### 2.8 Immunofluorescence

PDAC tissues were fixed with 4% paraformaldehyde (PFA) for 30 min and embedded in optimal cutting temperature (OCT) compound. The appropriate primary and secondary antibodies were used to perform immunostaining. Nuclei were counterstained with DAPI respectively. Images were taken with an SP‐8 confocal Microscope.

### 2.9 Immunohistochemistry staining on PDAC tissue microarrays

It was as previously described to get immunochemical staining.^22^ Tissue microarrays (TMAs) containing paraffin sections from 110 PDAC patients (in the year 2016–2017) from Ruijin Hospital were immunostained for CD68, CD206 and CTCF proteins. TMAs were fixed in 40% PFA overnight at 4°C and dehydrated by an alcohol gradient. Anti‐CTCF (3418; CST, USA), anti‐CD206 (24595; CST), and anti‐CD68 (76437; CST) were used as primary antibodies.

### 2.10 RNA‐fluorescence in situ hybridisation for LncRNA‐PACERR

It was as previously described for RNA‐fluorescence in situ hybridisation (RNA‐FISH).^23^ Diethyl pyrocarbonate was used to fix THP‐1 for more than 12 h. Next, THP‐1 were dehydrated by an alcohol gradient and embedded in paraffin. Fluorescence‐labelled single‐strand probes were hybridised. PACERR oligos were gotten from Servicebio Technology. After labelling, images were taken with a fluorescence microscope (Zeiss). The probe sequence of PACERR was TCTTCTGTCCCGACGTGACTTCCTCGACCCTCTA.

### 2.11 Tumour xenograft assay

A total of 1 × 10^7^ PANC‐1 WT cells and 5 × 10^6^ THP‐1 cells were resuspended in 30 μl of phosphate‐buffered saline (PBS) and were mixed and co‐injected into the spleen of 6‐week‐old BALB/c nude mice. BMDMs were isolated from C57BL/6 mice. Then BMDMs were transfected using siCTCF/siNC plasmids. Briefly, 1 × 10^7^ PAN02 WT cells and 5 × 10^6^ BMDMs (siCTCF/siNC) were co‐injected into the spleen of male 6‐week‐old C57BL/6 mice. The livers from each mouse were harvested after 2–4 weeks and embedded in paraffin for haematoxylin‐eosin (HE) staining.

### 2.12 RNA extraction, reverse transcription and qPCR analysis

Total RNA from TAMs, monocytes from patients’ peripheral blood and cell lines used in this study was extracted in TRIzol (Invitrogen, USA). RNA in the nucleus or cytoplasm was extracted with a PARIS Kit (Invitrogen). We reverse‐transcribed synthesized cDNA by using HiScript III‐RT SuperMix for quantitative polymerase chain reaction (qPCR) (+gDNA wiper; Vazyme Biotech, China). Relative RNA expression levels determined by reverse transcription‐qPCR (RT‐qPCR) were measured on a 96‐well plate by using AceQ Universal SYBR qPCR Master Mix (Vazyme Biotech); the related gene‐specific primers used are listed in Table S2. GAPDH, β‐actin and U6 were used as positive controls for PACERR and mRNA. Quantitation of expression of related genes was performed using the 2^–∆∆CT^ method.

### 2.13 Nuclear and cytoplasmic RNA isolation

A nuclear and cytoplasmic fractionation kit (Thermo AM1921) was used to isolate nuclear and cytoplasmic RNA according to the instructions of the manufacturer. LncRNA‐PACERR expression levels were detected by qPCR in both the cytoplasm and nucleus.

### 2.14 Western blot

Cells were lysed with WB/IP lysis buffer (Beyotime), and the split products were qualified by Bicinchoninic acid protein assay (Beyotime). Polyacrylamide gel electrophoresis Gel Fast Preparation Kits (EpiZyme) were used to prepare the gel for electrophoresis. Antibodies against CTCF (ab26271, Abcam, USA), COX2 (ab16895; Abcam, USA), Glyceraldehyde 3‐phosphate dehydrogenase (GAPDH, 3936; CST), β‐tubulin (2128; CST) and EP300 (ab275378; Abcam) were used. GAPDH was used as a protein of internal reference. The results were taken by Tanon‐5200 Chemiluminescent Imaging System.

### 2.15 Enzyme‐linked immunosorbent assay

The enzyme‐linked immunosorbent assay (ELISA) was performed as previously described.^24^ Human IL10 ELISA kit, Human TGFβ ELISA kit and Human Arginase‐1 ELISA kit were used to detect the concentrations of IL10, TGFβ and Arginase‐1 which were performed following the manufacturer's instructions in undiluted supernatants from THP‐1 cells co‐cultured with tumour cells in 6‐well‐plates.

### 2.16 Luciferase reporter assays

THP‐1 cells co‐cultured with PANC‐1 or PATU‐8988 WT were in a 12‐well‐plate at 1 × 10^5^ cells per well overnight. After 24 h, THP‐1 were co‐transfected with PACERR‐OE and PTGS2–promoter‐reporter plasmids (Genechem, Shanghai). After 24 h, Cell lysis buffer (Vazyme Biotech) was used to lyse THP‐1, and the luciferase activity was measured by a Microplate Reader (BioTek) was used to measure the luciferase activity which was normalised to the luciferase activity of renilla.

### 2.17 Flow cytometry

Cells were resuspended in 50 μl of staining buffer (PBS:FBS = 1000:1) with 2 μl of Fc block (422302; Biolegend, USA) for 15 min at 4°C. Then, 1.5 μl of anti‐CD163‐PE (333605; Biolegend) and 1.5 μl of anti‐CD206‐APC (321109; Biolegend) were added to the reaction for 30 min at 4°C. After 30 min, THP‐1 were washed twice with staining buffer, and 1% formaldehyde was used for fixation at 4°C. The data were detected by flow cytometry (Beckman Coulter).

### 2.18 RNA‐sequencing

The RNA‐sequencing (RNA‐seq) assay was performed as previously described.^25^ RNeasy Mini Kit (QIAGEN, Germany) was used to purify RNA products (QIAGEN). Note that, 2 × 150 paired‐end sequencing was performed for analysis.

### 2.19 Assay for transposase‐accessible chromatin with sequencing

The assay for transposase‐accessible chromatin with sequencing (ATAC‐seq) assay was performed as previously described.^26^ One million fresh cells (THP‐1 cells cocultured with PANC‐1 cells treated with PMA, TAMs and monocytes) were lysed in 100 μl of lysis buffer per sample for 15 min on ice to obtain the nuclei briefly. Immediately, nuclei were centrifuged at 500 x g for 5 min. Then, nuclei were incubated at 37°C with tagmentation buffer and Tn5 transposome (Vazyme Biotech) for 30 min. Note that, 2 × 150 paired‐end‐sequencing was performed for analysis.

### 2.20 Chromatin immunoprecipitation

The chromatin immunoprecipitation (ChIP) assay was performed as previously described.^27^ H3K27ac (ab4729; Abcam), H3K27me3 (9733S; CST), CTCF (07–729; Millipore), H3K9ac (9649; CST), H3K4me (5326; CST) and EP300 (ab275378; Abcam) were used for ChIP experiments. For ChIP‐sequencing (ChIP‐seq), 10–50 ng product was used to generate the DNA library using a VAHTS Universal DNA Library Prep Kit for Illumina V3 (Vazyme Biotech). The ChIP DNA Library was sequenced with Illumina HiSeq X Ten. For ChIP‐qPCR, ChIP products that were used for RT‐PCR were used to amplify the PCR products for 45 cycles.

### 2.21 Chromatin isolation through RNA purification with sequencing

The chromatin isolation through RNA purification with sequencing (ChIRP‐seq) assay was performed as previously described.^28^ 1% PFA was used to crosslink 1 × 10^8^ THP‐1 at room temperature on a sky wheel for 30 min. Note that, 2.5 mM glycine was added to end the reaction and the solution was centrifuged at 800 x g for 5 min.

### 2.22 RNA immunoprecipitation

We used a MagnaRIP RNA‐Binding Protein Immunoprecipitation Kit (Millipore) for RNA immunoprecipitation (RIP) experiments. The antibodies were purchased from Millipore (CTCF 07–729), Elabscience (IgG E‐AB‐1034) and CST (FLAG 14793). The qPCR primers are listed in Table S1.

### 2.23 Co‐immunoprecipitation

Note that, 500 μl of IP lysis buffer (Beyotime) with Proteinase Inhibitor (NCM Biotech) was used to lyse 1 × 10^7^ cells for 10 min on ice. Nuclear extracts were centrifuged at 14 000 rpm for 20 min at 4°C. After 15 min, 1 μg of anti‐CTCF/anti‐EP300 suspended in lysis buffer was added to the supernatant, which was collected in an EP tube. Then, this EP tube was incubated on a sky wheel for 1 h. After 1 h, the reaction mixtures were incubated with 25 μl of protein A Dynabeads (Invitrogen) at 4°C overnight. The products of immunoprecipitation were washed six times with IP lysis buffer. The products were added to the loading buffer and put in a metal bath at 100°C for 15 min. Then, the beads were washed and recovered using magnets.

### 2.24 Statistical analysis and data visualisation

GraphPad Prism 8 and the R platform were used for statistical analysis. Three to five biological replicates were showed where pointed. Data are expressed as the mean ± standard deviation, and the difference between the two groups was calculated by the unpaired two‐tailed Student's *t*‐test.

## 3 RESULTS

### 3.1 CTCF was highly expressed and associated with hyper‐accessible chromatin regions in TAMs

**FIGURE 1:**
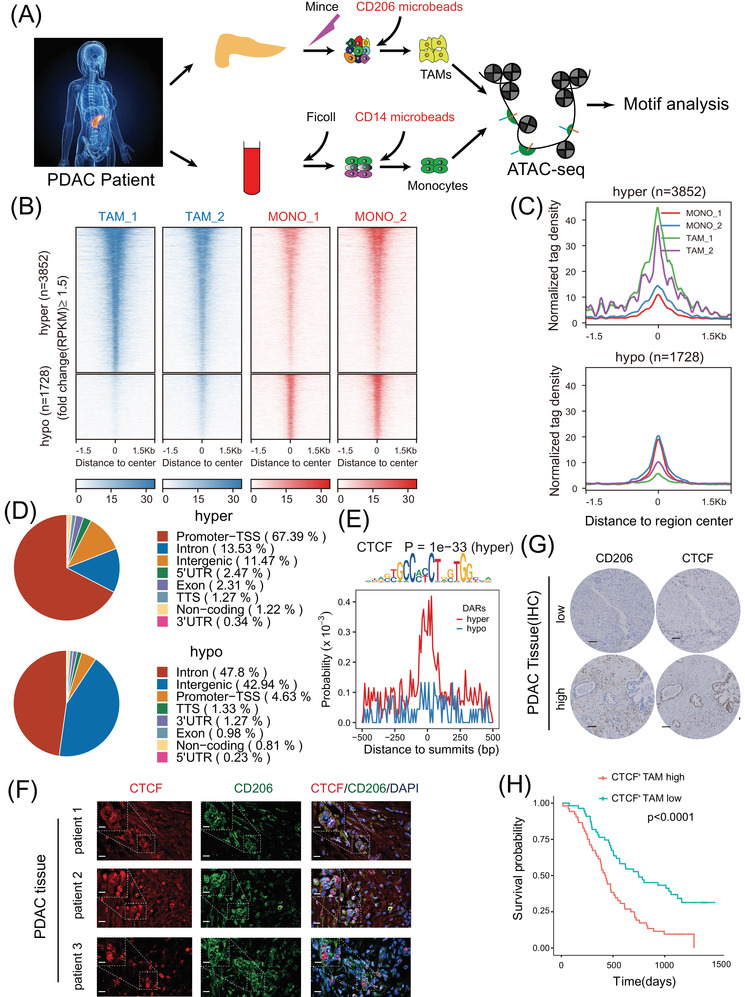
Assay for transposase‐accessible chromatin with sequencing (ATAC‐seq) reveals CCCTC‐binding factor (CTCF) as a key transcription factor in tumour‐associated macrophages (TAMs) (A) Schematic illustration of ATAC‐seq two sample preparation (*n* = 2). Tumour‐associated macrophages (TAMs) from two pancreatic ductal adenocarcinoma (PDAC) tissue samples were enriched with CD206 positive selection. Monocytes (MONO) from blood samples of the same patient were enriched with CD14 positive selection. (B) Heatmap showing normalised ATAC‐seq signals (RPKM) in TAMs and monocytes (MONO) over differentially accessible regions (DARs). The top set of panels shows read signals over the 3852 hyper‐accessible regions, while the bottom set of panels shows read signals over the 1728 hypo‐accessible regions in TAMs. Signals within 1.5 kb surrounding the centre of DARs are displayed in descending order. (C) Profiles of normalised tag density across a genomic window of ±1.5 kb surrounding the centre of hyper‐ and hypo‐accessible regions. (D) Pie‐chart showing the proportion of hyper‐ and hypo‐accessible sites within the indicated genomic regions: exons, intergenic regions, introns, 3′ UTR, 5′ UTR, promoters‐transcription start site (TSS), TES and noncoding regions. Peak summits located up to 1 kb upstream and 100 bp downstream of the TSS were determined to be promoter‐TSS regions. (E) Distribution probability of CTCF binding motifs around ATAC‐seq peak summits in DARs. The *p*‐value was calculated by HOMER for the CTCF motif enriched in hyper‐accessible regions. (F) Colocalisation of CTCF (red) and CD206 (green) in three clinical samples of pancreatic ductal adenocarcinoma (PDAC) as shown by fluorescence microscopy. DAPI staining (blue) shows the nuclei (DNA). Scar bar: up panel, 15 μm; down the panel, 50 μm. (G) Representative IHC images of serial PDAC tissues stained for CD206 and CTCF. The 110 PDAC patients were divided equally into two groups (“low” and “high”) based on the median percentage of CTCF^+^ TAM infiltration in the tumour microenvironment. Scar bar: 200 μm. (H) Kaplan‐Meier survival curve presenting the overall survival of 110 PDAC patients, grouped according to the extent of CTCF^+^ TAM infiltration

To characterise the accessible chromatin landscape in TAMs, we used MACS to isolate high‐purity CD206^+^ cells which were indeed macrophages from tumour tissues and high‐purity CD14^+^ cells from peripheral blood from PDAC patients and compared the genome‐wide maps of ATAC‐seq between CD206^+^ TAMs and CD14^+^ monocytes (Figure 1A and Figure S1A–F). In total, 3852 regions were ≥1.5‐fold more accessible in TAMs (defined as hyper‐accessible regions), while 1728 regions were ≥1.5‐fold more accessible in monocytes (defined as hypo‐accessible regions) (Figure 1B). TAMs were associated with higher tag densities over hyper‐accessible regions, while monocytes were associated with higher tag densities over hypo‐accessible regions, suggesting the increased global transcription activity of TAMs (Figure 1C). Most hyper‐accessible sites were enriched in promoter transcription start sites (TSSs), while hypo‐accessible sites were most frequently distributed in intronic and intergenic regions (Figure 1D).

Chromatin accessibility regulates cellular transcriptional states by affecting the binding of transcription factors (TFs). To identify the key transcription factors linking the chromatin accessibility with transcriptional characteristics of TAMs, we performed sequence‐based motif analysis with HOMER v4.9^29^ and found that the binding sites of CTCF which was a high rank of TFs by motif analysis were significantly enriched in hyper‐accessible regions (Figure 1E) and some previously published articles revealed that CTCF critically controls gene expression in macrophages via epigenetic regulation,^30^, ^31^ indicating the upregulation of CTCF activity in TAMs. CTCF was a heritable component of an epigenetic system regulating the interplay between DNA methylation, high chromatin structure, and lineage‐specific gene expression, so we were interested in studying the function of CTCF and locating the key CTCF binding sites in TAMs.

### 3.2 Higher infiltration of CTCF^+^ TAMs is associated with worse prognosis in PDAC

The RNA and protein levels of CTCF in CD206^+^ TAMs, CD14+ monocytes and CD11b^+^ normal tissue‐resident macrophages of which the purity was 87.5% after CD11b sorting were validated by RNA sequencing, qPCR, western blot (Figure S2A–E). Interestingly, CTCF was mainly expressed in the nucleus of CD206^+^ TAMs by IF analysis (Figure 1F and Figure S2F), consistent with its role as an epigenetic regulator. To further investigate the prognostic value of CTCF^+^ TAMs, we performed immunohistochemistry (IHC) staining of CTCF and CD206 on serial sections from TMAs of 110 PDAC patients (Table 1 and Figure 1G). The clinical characteristics of these patients (64 males and 46 females) whose average age is 62.8 are shown in Table 1. After 110 fields were analysed, the PDAC patients were divided into two groups based on the median density of CTCF^+^ TAM infiltration (the percentage of CTCF^+^ TAMs number in total CD206^+^ cells number) in the tumour microenvironment in 110 PDAC patients. Interestingly, Kaplan‐Meier analysis of overall survival (OS) through IHC (Figure 1H) and IF (Figure S2G) of TMAs revealed that higher infiltration of CTCF^+^ TAMs was associated with worse prognosis in PDAC, demonstrating the clinical relevance of CTCF activity in TAMs.

**TABLE 1:** Clinical characteristics of 110 pancreatic cancer patients in tissue microarray

Variable	Value	*p*‐value (CTCF+ TAM high vs. low)
Age	62.8 ± 9.5	.0024
Sex (male)	64 (58.2%)	.132
WBC	5.55 (4.53, 6.7)	.164
Hb	131 (119, 137)	.425
PLT	183 (138, 239)	.237
Tbil (Total bilirubin)	19.9 (13.4, 106.6)	.014
Dbil (Direct bilirubin)	3.4 (1.95, 59.7)	.028
Fasting glucose	6.14 (5.38, 8.03)	.184
CA125	18.55 (11.4, 32.28)	.005
CA199	149.9 (49.6, 350.05)	.0042
CEA	3.64 (2.28, 7.75)	.0073
Tumour location		
Head	72 (65.5%)	.326
Body/tail	38 (34.5%)	.373
Surgery		
PD	72 (65.5%)	.733
DP	38 (34.5%)	.568
T stage		
T1	11 (10%)	.004
T2	62 (56.4%)	.007
T3	18 (16.4%)	.003
T4	18 (16.4%)	.009
N stage		
N0	50 (45.5%)	.005
N1/N2	60 (54.5%)	.017
M stage		
M0	103 (93.6%)	.026
M1	7 (6.4%)	.034
American Joint Committee on Cancer (AJCC) stage		
I	31 (28.2%)	.036
II	50 (45.5%)	.042
III	22 (20%)	.021
IV	7 (6.4%)	.026
Grade		
Low	87 (79.1%)	.017
Median/high	23 (20.9%)	.029

### 3.3 CTCF is a critical regulator that promotes the M2 polarization and pro‐tumour functions of macrophages

**FIGURE 2:**
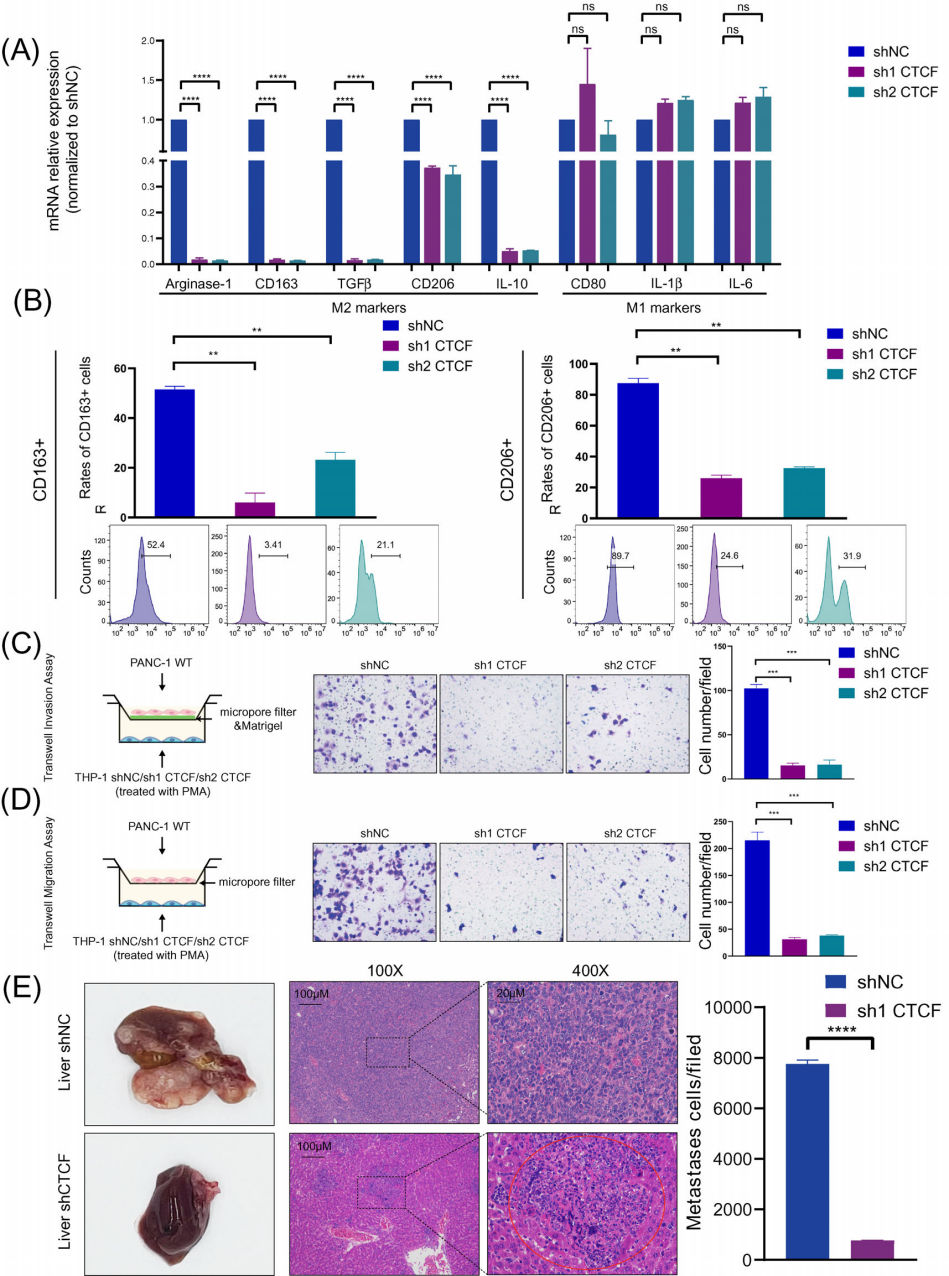
Knockdown of CCCTC‐binding factor (CTCF) hinders the M2 polarization and pro‐tumour functions of THP‐1‐derived tumour‐associated macrophages (TAMs). (A) Quantitative polymerase chain reaction (qPCR) analysis of the relative expression of M2 markers (Arginase‐1, CD163, TGFβ, CD206, and IL‐10) and M1 marker (CD80, IL‐1β and IL‐6) in THP‐1‐derived TAMs after CTCF knockdown. THP‐1 cells were treated with phorbol 12‐myristate 13‐acetate (PMA) and co‐cultured with PANC‐1 cells for 2 days. Data are shown as the results from three independent experiments. (B) Flow cytometry analysis of the expression of M2 markers (CD163 and CD206) in THP‐1‐derived TAMs after CTCF knockdown. THP‐1 cells were treated with PMA and co‐cultured with PANC‐1 cells for 2 days. Data are shown as the results from two independent experiments. (C) Invasion capacity of PANC‐1 cells co‐cultured with THP‐1‐derived TAMs (shNC/ shCTCF). shNC means that cells were transfected in negative control plasmids. (D) Migration capacity of PANC‐1 cells co‐cultured with THP‐1‐derived TAMs (shNC/ shCTCF). (E) Representative images of liver metastasis and the number of metastatic cells in the pancreatic ductal adenocarcinoma (PDAC) mouse model, in which PANC‐1 cells mixed with TAMs (THP‐1 shNC/sh1 CTCF) were injected into the spleens of BALB/c nude mice. Data are shown as the results from three independent experiments. ^*^ *p* < .05; ^**^ *p* < .01; ^***^ *p* < .001; ^****^ *p* < .0001

To investigate the impact of CTCF on the phenotype and function of TAMs, we conducted stable knockdown of CTCF expression in THP‐1 cells before differentiation towards macrophages using lentivirus‐mediated shRNAs (MOI: 100) against CTCF (Figure S3A,B) and used CCK8 assay to find that the viability of THP‐1 was no difference between WT group and CTCF knockdown group (Figure S3C,D), and established in vitro TAM models by differentiating THP‐1 monocytes into macrophages with PMA, and co‐culturing them with PDAC cell lines PANC‐1 and PATU‐8988. Interestingly, qRT‐PCR revealed that the expression of M2 differentiation markers Arginase‐1, CD163, TGFβ, CD206 and IL‐10 were downregulated in CTCF knockdown TAMs and the expression of classical M1 markers CD80, IL‐1β and IL‐6 were not affected (Figure 2A). Flow cytometric analyses confirmed that depletion of CTCF expression in TAMs resulted in the significantly diminished percentages of CD163^+^ and CD206^+^ M2 macrophages (Figure 2B). Besides, M2‐secreted IL‐10, Arginase‐1 and TGFβ were also decreased in CTCF knockdown TAMs by ELISA (Figure S4A). According to our previous report, TAMs, especially M2 macrophages, could promote PDAC metastasis through epithelial‐mesenchymal transition (EMT).^32^ Therefore, we wondered whether the knockdown of CTCF in TAMs could hinder this effect. To address this question, we investigated the influence of CTCF knockdown in TAMs on migration and invasion ability of PDAC cell lines based on in vitro co‐culture system and Transwell assays (Figure 2C,D and Figure S4B,C). As expected, the PDAC cells co‐cultured with CTCF knockdown TAMs presented with the significantly suppressed ability of invasion (Figure 2C,D and Figure S4B,C). We further assessed the impact of CTCF expression in macrophages on supporting tumour metastasis in a PDAC mouse model of liver metastasis, in which PANC‐1 cells mixed with CTCF knockdown or control macrophages (THP‐1 shNC/sh1 CTCF cells) were injected into the spleens of BALB/c nude mice. The polarization of BMDMs was not changed after interfering with CTCF (Figure S4D,E). Besides, the spleens of C57BL/6 mice were injected PANC02 cells and BMDM cells (BMDM siNC/siCTCF cells) which were interfered with CTCF and were not co‐cultured with tumour cells before injecting to examine the impact of CTCF (Figure S4D). To determine the role of CTCF in the polarization and infiltration of M2 phenotype macrophages, we performed IHC of CD68 (macrophage marker) and CD206 (M2 marker) in the liver tissue of shNC and sh1 CTCF mice (Figure S4F). We observed significantly fewer metastatic cells and a decrease in the number of CD206^+^ cells other than CD206^+^ sinusoidal endothelial cells in metastatic foci and no changes in the number of CD68^+^ cells which may include CD68^+^ Kuppffer cells in metastatic foci in the livers of mice receiving CTCF knockdown macrophages in comparison to control groups (Figure 2E and Figure S4G). Interestingly, the co‐injection of CTCF knockdown macrophages can't inhibit the formation of the basal level of metastasis. (Figure S4H). And similar results were validated in these findings through C57BL/6 mice indicate that CTCF is a critical regulator promoting the M2 polarization and pro‐tumour functions of macrophages (Figure S5A–C).

### 3.4 Integrative transcriptome and epigenome analysis revealed PTGS2 and PACERR as downstream targets of CTCF in TAMs

**FIGURE 3:**
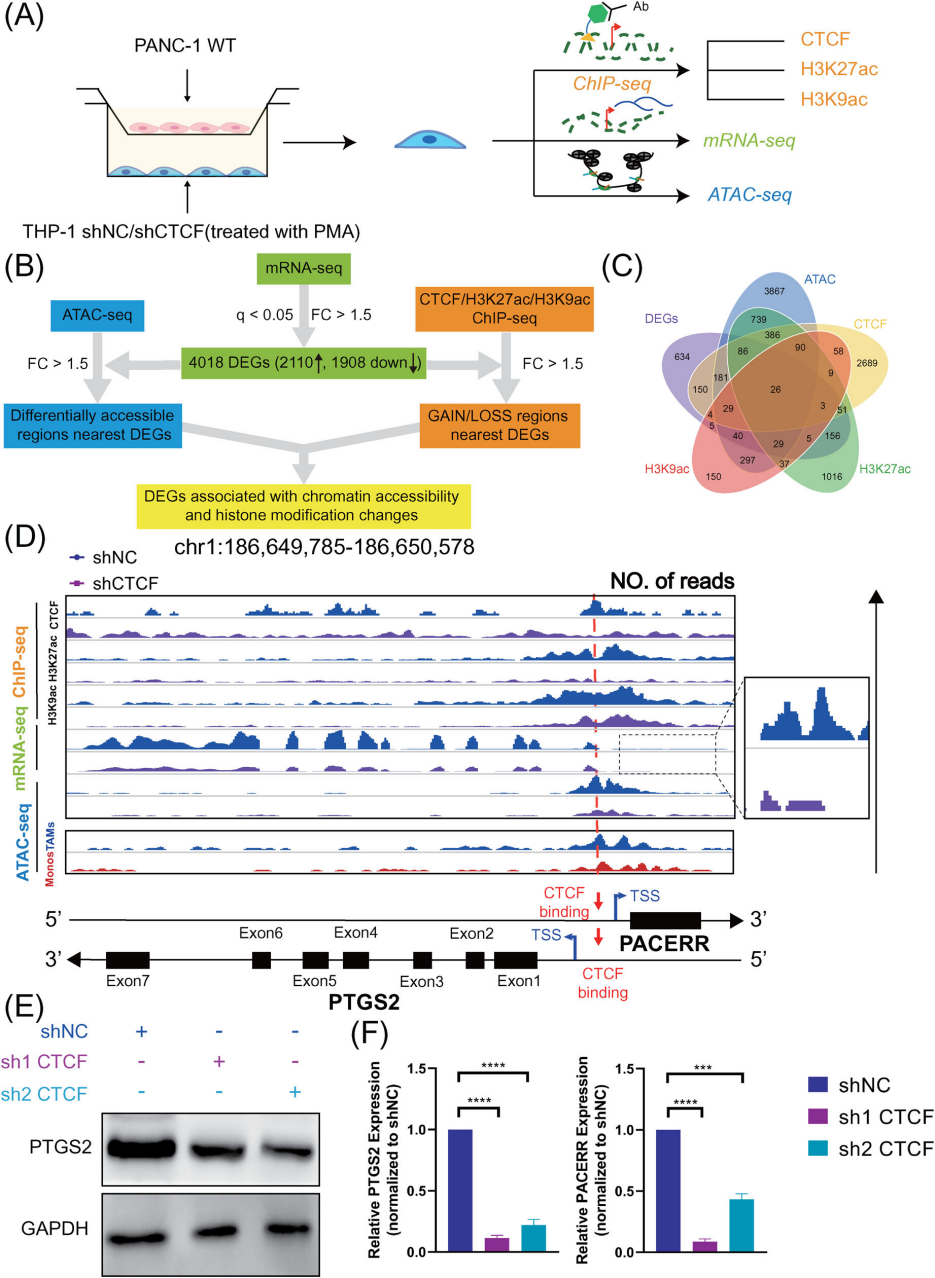
Integrative multi‐omics analysis and experimental validation reveal that prostaglandin‐endoperoxide synthase 2 (PTGS2) and PTGS2 antisense NF‐κB1 complex‐mediated expression regulator RNA (PACERR) are downstream targets of CCCTC‐binding factor (CTCF) in THP‐1‐derived tumour‐associated macrophages (TAMs). (A) Graphical scheme describing the workflow of performing RNA‐seq, ChIP‐seq and ATAC‐seq on control and CTCF‐knockdown THP‐1‐derived TAMs (THP‐1 stimulated with PMA and co‐cultured with PANC‐1). (B) Graphical scheme describing the workflow of comparing the transcriptome and epigenome between control and CTCF‐knockdown THP‐1‐derived TAMs (THP‐1 stimulated with phorbol 12‐myristate 13‐acetate [PMA] and co‐cultured with PANC‐1). (C) Venn diagram of the differentially expressed genes (DEG) results. (D) Genome browser snapshots of ChIP‐seq/mRNA‐seq/ATAC‐seq signals for the genomic regions near PTGS2 and PACERR in THP‐1‐derived TAMs (shNC/ shCTCF THP‐1 stimulated with PMA and co‐cultured with PANC‐1). shNC means that cells were transfected in negative control plasmids. ATAC‐seq signals of TAMs and monocytes (MONO) from pancreatic ductal adenocarcinoma (PDAC) clinical samples were also visualised. (E) PTGS2 protein expression in THP‐1‐derived TAMs (shNC and shCTCF THP‐1 stimulated with PMA and co‐cultured with PANC‐1), examined by Western blot. (F) PTGS2 and PACERR mRNA expression in THP‐1‐derived TAMs (shNC and shCTCF THP‐1 stimulated with PMA and co‐cultured with PANC‐1), examined by qPCR analysis. Data are shown as the results from three independent experiments. The image is representative of three independent experiments. ^*^ *p* < .05; ^**^ *p* < .01; ^***^ *p* < .001; ^****^ *p* < .0001. Abbreviations: DEG: differentially expressed genes; FC: fold change, q: *q*‐value

CTCF serves as a key epigenetic regulator in a chromosomal organisation and transcriptional regulation.^33^, ^34^ To investigate how CTCF regulates TAMs by profiling chromatin accessibility and histone modification changes, PANC‐1‐cocultured TAMs derived from WT and CTCF knockdown THP‐1 cells were performed to multi‐omics analysis (Figure 3A). First, we performed RNA‐seq analysis to compare the expression profiles of CTCF shRNA and control shRNA transfectants (raw data accessible via GSE169451) (Figure S6A–E) and identified 2110 upregulated genes and 1908 downregulated genes (|Fold Change| > 1.5, *q* < .05) after knockdown of CTCF in TAMs (Figure 3B). Next, according to CTCF chromatin immunoprecipitation (ChIP)‐seq assay (THP‐1 shNC/shCTCF, GS169451), 479 differentially expressed genes were directly bound by CTCF (Figure S7A–D). A majority of CTCF binding sites were distributed in the intergenic regions, indicating that CTCF may control gene expression via long‐range interactions between promoters and enhancers^35^ (Figure S7C). In order to reveal the direct transcriptional regulation of those genes by CTCF, we focussed on the CTCF binding sites which were distributed near the TSS region. We further investigated the influence of CTCF on the chromatin status using ATAC‐seq (THP‐1 shNC/shCTCF, GSE169451) (Figure S8A–D) and ChIP‐seq assays of H3K27ac, H3K9ac and H3K27me3 (THP‐1 shNC/shCTCF, GSE169451) (Figures S9A‐D, S10A–D and S11A–C). Among the 479 genes selected above, we identified 26 genes with altered chromatin accessibility after knockdown of CTCF (Figure 3C and Figure S12A,B), demonstrating the epigenetic regulation of CTCF on the expression of those genes.

Compared with other candidate genes such as PPIF, SRGN and AGO2, PTGS2 (COX‐2), one of the famous genes selected as above with most essential in the M2 polarization and pro‐tumour function of TAMs cells in previous reports, was chosen as a typical example for analyzing the mechanism of CTCF acting as a transcription factor in tumorigenesis (Figure 3D and Figure S12B).^36^, ^37^, ^38^, ^39^, ^40^, ^41^ A CTCF peak was identified within the PTGS2 promoter in WT THP‐1‐derived TAMs, while not in CTCF knockdown TAMs (Figure 3D). To verify the regulatory role of CTCF on PTGS2 expression in TAMs, we examined the expression of PTGS2 after CTCF knockdown in THP‐1‐derived in vitro TAM model and analysed the chromatin modification status in PTGS2 promotor (Figure 3E,F and Figure S13A–C). Consistent with bioinformatics analysis, the expression of PTGS2 was downregulated after CTCF knockdown on both mRNA and protein levels (Figure 3E,F). CTCF binding to the promoter region of PTGS2 was disrupted after CTCF knockdown, accompanied by decreased enrichment of H3K27ac and H3K9ac (Figure S13A), while the enrichment of H3K27me3 and H3K4me1 was not changed significantly (Figure S13B,C).

Of note, PACERR, an antisense LncRNA, is located just –.3 kb upstream of the PTGS2 mRNA start site. Given that the promoter region of PACERR exhibits substantial overlap with the promoter of PTGS2, and PACERR is transcribed in the opposite direction to PTGS2 (Figure 3D), we anticipated that CTCF would also regulate the expression of PACERR in a manner similar to that of PTGS2 mRNA. This hypothesis was validated by qPCR results demonstrating the decreased expression of PACERR after CTCF knockdown in THP‐1‐derived TAMs (Figure 3F).

Interestingly, chromatin accessibility near the TSS region of PTGS2 was increased in TAMs compared to peripheral blood monocytes according to the ATAC‐seq of PDAC clinical samples (Figure 3D and Figure S14A,B).

In summary, based on multi‐omics analysis, experiment validation and the association between CTCF levels and PTGS2 or PACERR levels in TAMs of clinical samples (Figure S14C,D). PTGS2 and PACERR are potential downstream targets of CTCF in TAMs.

### 3.5 PACERR positively regulates PTGS2 expression through binding to the promoter region

**FIGURE 4:**
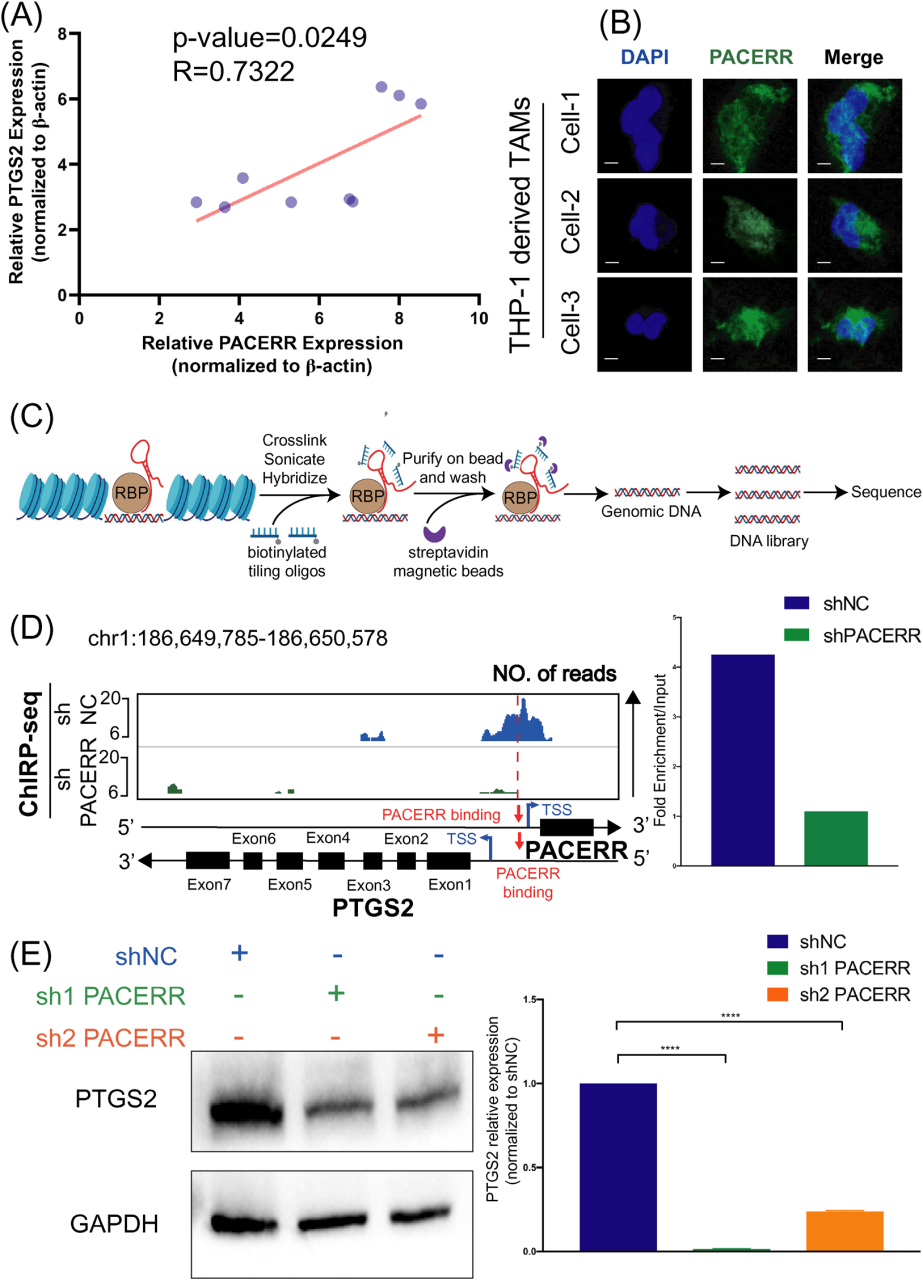
PTGS2 antisense NF‐κB1 complex‐mediated expression regulator RNA (PACERR) binds to the promoter region of prostaglandin‐endoperoxide synthase 2 (PTGS2) and positively regulates PTGS2 expression. (A) Linear regression and Spearman correlation analysis of the relative expression of PACERR and PTGS2 in tumour‐associated macrophages (TAMs) isolated from pancreatic ductal adenocarcinoma (PDAC) tissues (*n* = 9). (B) Fluorescence in situ hybridisation (FISH) of PACERR (green) in THP‐1‐derived TAMs. DAPI staining (blue) shows the nuclei. Scar bar: 10 μm. (C) Graphical scheme describing the workflow of chromatin isolation through RNA purification with sequencing (ChIRP‐seq) experiment. (D) Genome browser snapshots of ChIRP‐seq signals for the genomic regions near PTGS2 and PACERR in THP‐1‐derived TAMs (shNC/shPACERR, stimulated with phorbol 12‐myristate 13‐acetate [PMA] and co‐cultured with PANC‐1). shNC means that cells were transfected in negative control plasmids. The PACERR binding sites are shown as dashed lines. The histogram displays ChIRP‐seq signals (fold enrichment over input) of PACERR near PTGS2 promoter regions in shNC and shPACERR THP‐1‐derived TAMs. (E) PTGS2 protein and mRNA expression examined by Western blot assay and qPCR analysis, respectively, in THP‐1‐derived TAMs (shNC/ shPACERR, stimulated with PMA and co‐cultured with PANC‐1)

Cis‐regulation was an attributed function of LncRNAs.^42^ Given that PACERR is transcribed in the opposite direction to the nearby PTGS2 gene, in line with the characteristics of the distance from the targets of *cis*‐acting LncRNAs in the linear genome (Figure 3D), we asked whether PACERR might participate in *cis*‐regulation of PTGS2 transcription. TAMs were sorted from nine PDAC patients and linear regression and Spearman correlation analysis of the relative expression of PACERR and PTGS2 showed that they were positively correlated (Figure 4A). First, we examined the subcellular distribution of PACERR in TAMs using cellular fractionation assays and FISH, which revealed the abundant distribution of PACERR in the nucleus and cytoplasm in THP‐1‐derived TAMs (Figure 4B and Figure S15A). Here we focussed on the mechanism of the nuclear fraction of PACERR, suggesting that PACERR may exert at least part of its biological function in the nucleus. Next, we performed ChIRP‐seq for mapping of PACERR binding sites on the chromosome (Figure 4C and Figure S15B–D). ChIRP‐seq revealed that PACERR could bind to PTGS2 promoter (Figure 4D), while knockdown of PACERR significantly reduced this interaction (Figure 4D). And the interaction between PACERR and PTGS2 promoter were elucidated in dual luciferase report assay (Figure S15E). In addition, the mRNA and protein levels of PTGS2 in PACERR knockdown THP‐1‐derived TAMs were significantly lower compared with control TAMs (Figure 4E and Figure S16A). Interestingly, the expression of PACERR and PTGS2 in sham group macrophages was very low and upregulated after THP‐1 co‐culture with pancreatic cancer cells (Figure S16A,B). It means that PACERR and PTGS2 may be more tumour‐specific therapeutic targets. Taken together, these findings demonstrated that PACERR functions as a nuclear LncRNA that binds to the promoter region of PTGS2 and positively regulates PTGS2 expression.

### 3.6 PACERR is essential for the M2 polarization and pro‐tumour functions of TAMs

**FIGURE 5:**
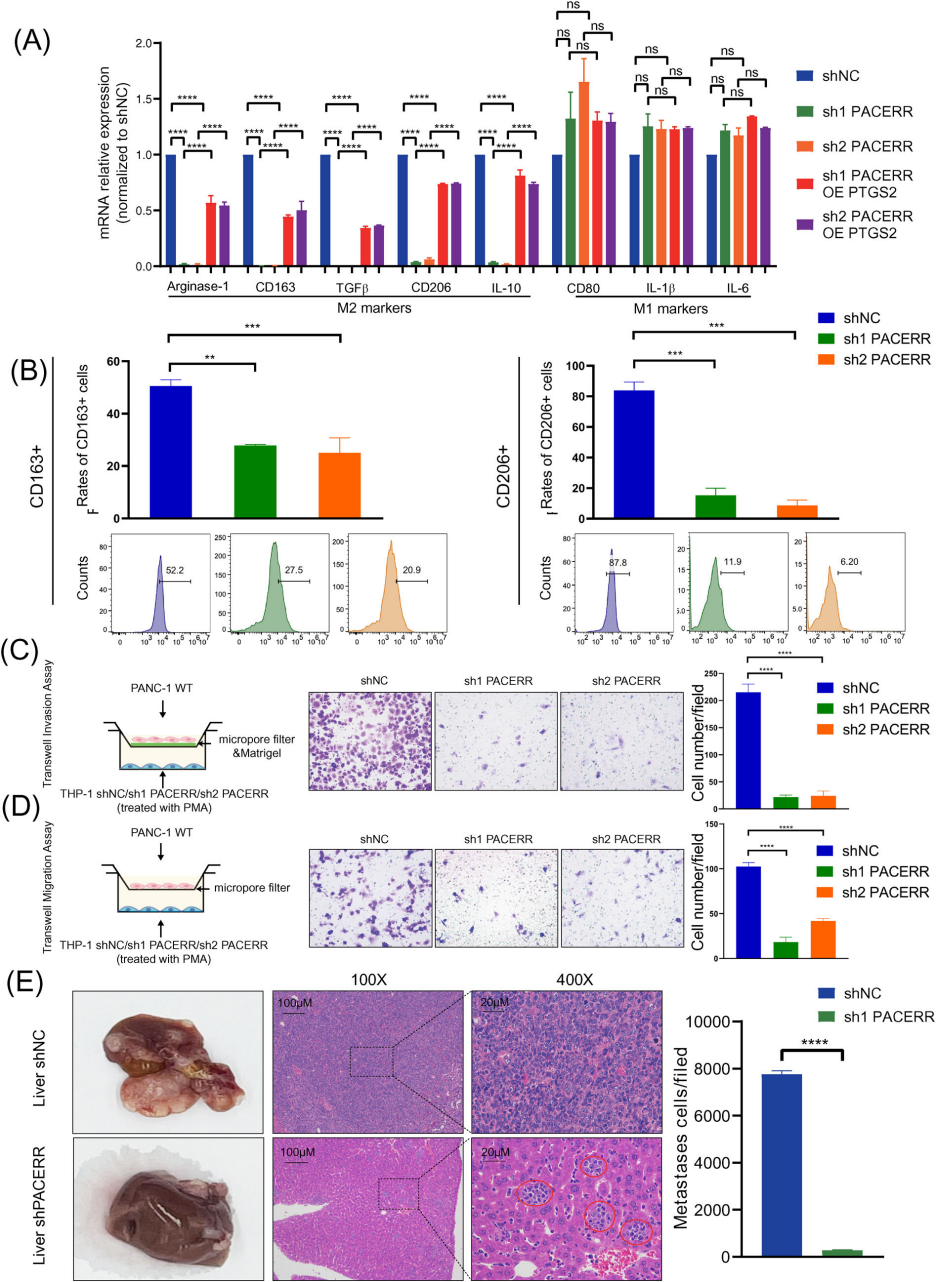
PTGS2 antisense NF‐κB1 complex‐mediated expression regulator RNA (PACERR) knockdown hinders the M2 polarization and pro‐tumour functions of THP‐1‐derived tumour‐associated macrophages (TAMs). (A) Quantitative polymerase chain reaction (qPCR) analysis of the relative expression of M2 markers (Arginase‐1, CD163, TGFβ, CD206, and IL‐10) and an M1 marker (CD80, IL‐1β, IL‐6) in THP‐1‐derived TAMs after PACERR knockdown or prostaglandin‐endoperoxide synthase 2 (PTGS2) overexpression. THP‐1 cells were treated with PMA and cocultured with PANC‐1 cells for 2 days. Data are shown as the results from three independent experiments. (B) Flow cytometric analysis of the expression of M2 markers (CD163 and CD206) in THP‐1‐derived TAMs after PACERR knockdown. THP‐1 cells were treated with phorbol 12‐myristate 13‐acetate (PMA) and cocultured with PANC‐1 cells for two days. Data are shown as the results from three independent experiments. (C) Invasion capacity of PANC‐1 cells co‐cultured with THP‐1‐derived TAMs (shNC/ shPACERR). shNC means that cells were transfected in negative control plasmids. (D) Migration capacity of PANC‐1 cells co‐cultured with THP‐1‐derived TAMs (shNC/ shPACERR). (E) Representative images of liver metastasis and the number of metastatic cells in the PDAC mouse model, in which PANC‐1 cells mixed with TAMs (shNC/sh1 PACERR THP‐1) were injected into the spleens of BALB/c nude mice. Data are shown as the results from three independent experiments. ^*^ *p* < .05; ^**^ *p* < .01; ^***^ *p* < .001; ^****^ *p* < .0001

**FIGURE 6:**
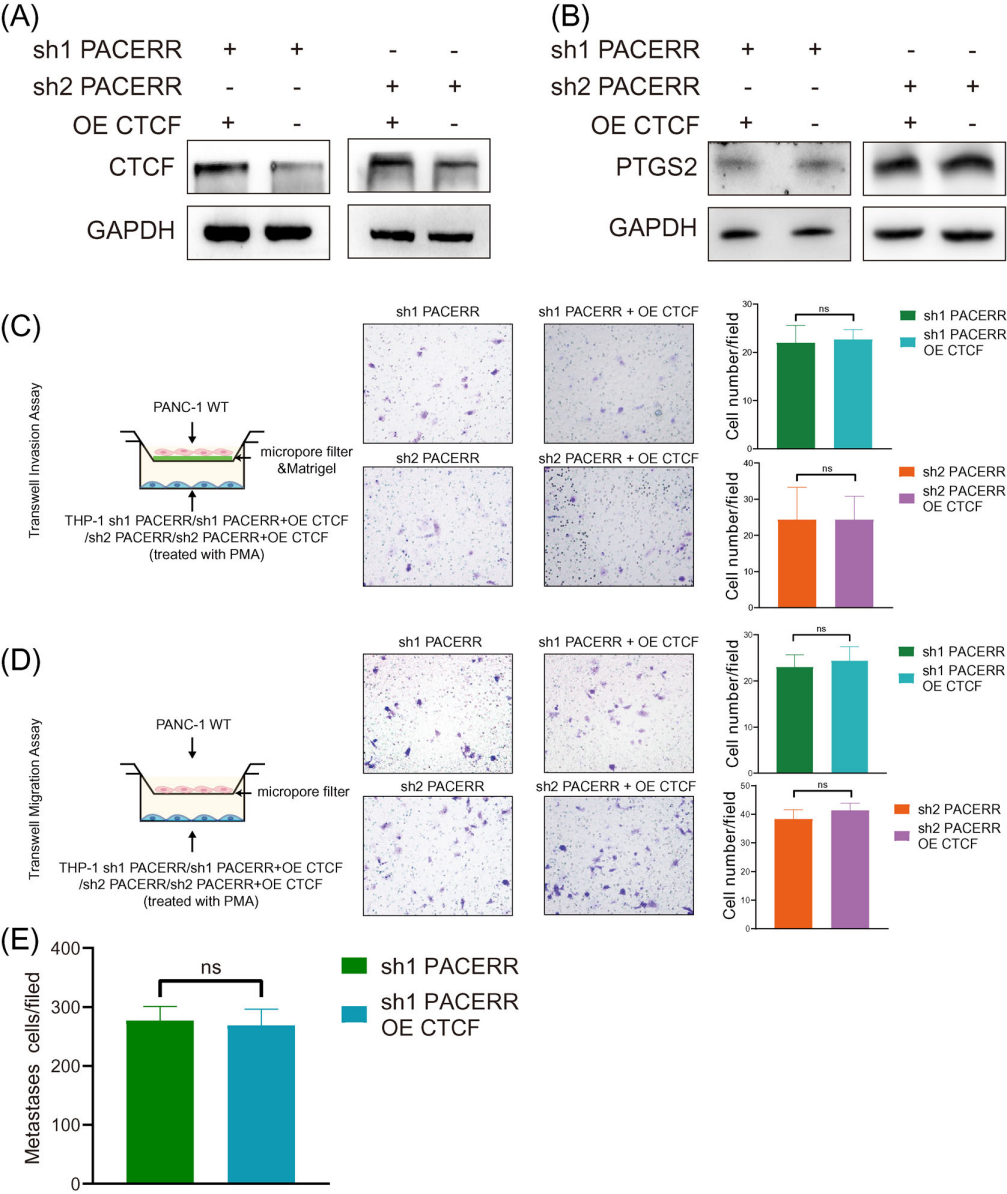
PTGS2 antisense NF‐κB1 complex‐mediated expression regulator RNA (PACERR) is necessary for the regulation of prostaglandin‐endoperoxide synthase 2 (PTGS2) in tumour‐associated macrophages (TAMs). (A,B) The protein expression of PTGS2 were not significantly changed after CCCTC‐binding factor (CTCF) overexpression in THP‐1‐sh1PACERR (A) derived TAMs and THP‐1‐sh2 PACERR (B) derived TAMs. (C) and (D) Invasion and capacity and migration capacity of PANC‐1 cells co‐cultured with THP‐1‐derived TAMs after CTCF overexpression in THP‐1‐sh1PACERR (C) derived TAMs and THP‐1‐sh2 PACERR (D) derived TAMs. “ns” means not statistically significant. Image is representative of three independent experiments

Next, we sought to explore whether PACERR could affect the phenotype and function of TAMs. PACERR stable knockdown THP‐1 monocytes were established using lentivirus‐mediated shRNAs against PACERR (Figure S16A) and converted into TAMs using the protocol as stated above. Similar to the effects of CTCF knockdown, qPCR, flow cytometry and ELISA analyses revealed that knockdown of PACERR decreased the expression levels of M2 differentiation markers, while the expression of classical M1 markers CD80, IL‐1β and IL‐6 remained unchanged (Figure 5A,B and Figure S17A). Consistent with these observations, THP‐1 cells in which PACERR was knocked down were overexpressed with PTGS2 (Figure S17B) and the M2 differentiation markers were significantly increased by macrophages transfected PTGS2 overexpression plasmid (Figure 5A,B and Figure S17C). Based on results of transwell assays, PDAC cells co‐cultured with PACERR knockdown TAMs showed significantly lower invasion and migration ability in comparison with those co‐cultured with control TAMs (Figure 5C,D and Figure S17D,E). Consistently, in the in vivo PDAC mouse models, mice injected with a mixture of PDAC cells and PACERR knockdown TAMs exhibited significantly reduced liver metastasis (Figure 5E), validating the essential role of PACERR in inducing pro‐tumour functions of TAMs. To further prove that PACERR is necessary for the regulation of PTGS2, we overexpressed CTCF in PACERR knockdown TAMs (Figure 6A). As expected, we found that the protein levels of PTGS2 were not significantly changed in CTCF‐overexpressed TAMs (Figure 6B). We used similar transwell models and BALB/c nude mice models to convince that these CTCF‐overexpressed TAMs did not increase the invasion and migration of pancreatic cancer cells in vitro and liver metastasis in vivo (Figure 6C‐‐E).

### 3.7 CTCF physically interacts with PACERR to recruit EP300 to the promoter region of PTGS2

**FIGURE 7:**
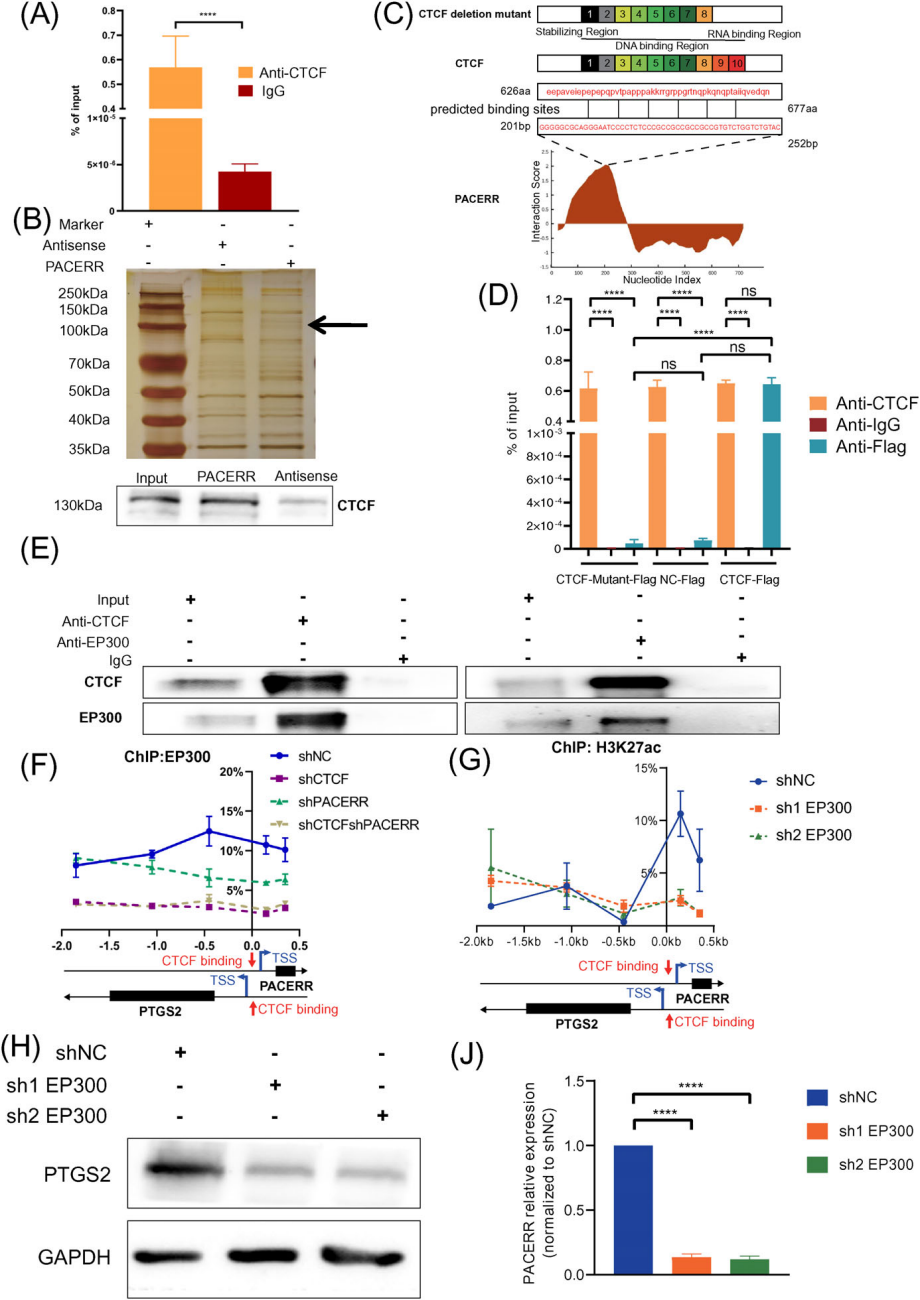
CCCTC‐binding factor (CTCF) binds directly to PTGS2 antisense NF‐κB1 complex‐mediated expression regulator RNA (PACERR) and recruits E1A binding protein p300 (EP300) to the promoter region of prostaglandin‐endoperoxide synthase 2 (PTGS2) in a PACERR‐dependent manner. (A) RNA immunoprecipitation (RIP) was performed using a CTCF‐specific antibody. Eluted CTCF‐binding RNAs were reverse transcribed, and quantitative polymerase chain reaction (qPCR) was performed with primers specific for PACERR. Normal rabbit IgG (IgG) was used as a negative control. Data are shown as the results from three independent experiments. (B) Imaging of RNA pull‐down experiment followed by silver staining. WB validation of CTCF proteins pulled down with PACERR is shown at the bottom. (C) Overview of CTCF domains and mutant control. The predicted RNA‐protein binding sites of PACERR and CTCF are shown at the bottom. (D) THP‐1 cells were infected with negative control‐Flag lentivirus or lentiviral virus encoding Flag‐tagged CTCF transcripts with or without the predicted RNA binding region (CTCF‐Flag or CTCF‐Mutant‐Flag) and stimulated into tumour‐associated macrophage (TAM) cell models before RIP assays. Whole‐cell lysates were subjected to immunoprecipitation with the indicated antibodies. Eluted RNAs were reverse transcribed, and qPCR was performed with primers specific for PACERR. Normal rabbit IgG was used as a negative control. (E) The results of co‐immunoprecipitation (Co‐IP) in THP‐1‐derived TAMs. Normal rabbit IgG was used as a negative control. (F) Association of EP300 with the promoter region of PTGS2 analysed by ChIP‐qPCR in THP‐1‐derived TAMs (shNC/ shCTCF/ shPACERR/ shCTCF & shPACERR). (G) Association of H3K27ac with the promoter region of PTGS2 and PACERR in THP‐1‐derived TAMs (shNC/ sh1 EP300/ sh2 EP300) analysed by ChIP‐qPCR. (H) Western blot analysis of PTGS2 protein expression in THP‐1‐derived TAMs after EP300 knockdown. (J) qPCR analysis of PACERR RNA level in THP‐1 derived TAMs after EP300 knockdown. The image is representative of three independent experiments. shNC means that cells were transfected in negative control plasmids. ^*^ *p* < .05; ^**^ *p* < .01; ^***^ *p* < .001; ^****^ *p* < .0001. “ns” means no statistically significance

It has been reported that CTCF‐RNA interactions might account for cell‐specific regulation of gene expression.^43^, ^44^ Our previous data demonstrated that CTCF and PACERR bind to the promoter region of PTGS2, and both of them are indispensable for the M2 polarization and pro‐tumour functions of TAMs. These findings prompted us to elucidate whether CTCF and PACERR form a complex to regulate PTGS2 expression. To test this hypothesis, we performed the RIP experiment, in which we immunoprecipitated endogenous CTCF from nuclear extracts of THP‐1‐derived TAMs, followed by extraction and analysis of the RNAs binding with CTCF. PACERR was detected in the immunoprecipitated product by qPCR (Figure 7A and Figure S18A). Reciprocally, RNA pull‐down and Western blot confirmed that CTCF was co‐precipitated with the biotin‐labelled PACERR, rather than biotin‐labelled antisense RNA (Figure 7B and Figure S18B). Next, we analysed the interaction between CTCF and PACERR using catRAPID omics.^45^ According to the algorithm, the most probable binding site of PACERR in CTCF was located in 626–677 aa sequence (Figure 7C). To validate the predicted binding site, we transfected THP‐1 cells with Flag‐tagged empty vector (NC‐Flag) or Flag‐tagged CTCF overexpression vector (CTCF‐Flag) or Flag‐tagged CTCF‐Mut vector (CTCF‐Mut‐Flag) in which the predicted binding sites were depleted (Figure 7D). Since CTCF‐Mut‐Flag was overexpressed in THP‐1 derived TAMs, Anti‐CTCF only pull‐down a little mutated protein and anti‐Flag merely pull‐down extremely few CTCF proteins in TAMs (Figure S18C). RIP analysis showed that deletion of the predicted RNA binding region significantly hindered the ability of CTCF to interact with PACERR compared to endogenous transcripts of CTCF (Figure 7D and Figure S18D), demonstrating that CTCF interacts directly with PACERR through its 626–677 aa sequence.

CTCF has been discovered to recruit histone modifiers, which regulates gene expression through alteration of the chromatin structure.^46^, ^47^, ^48^ Our previous data indicated that knockdown of CTCF mainly affected histone acetylation rather than histone methylation of the promoter region of PTGS2 (Figure S13A–C), suggesting that CTCF most likely recruited histone acetyltransferase (HAT) to the promoter region of PTGS2. Consistent with our speculation, co‐immunoprecipitation assays revealed the formation of a CTCF/EP300 complex in TAMs (Figure 7E and Figure S18E). Furthermore, we wondered whether the CTCF/PACERR complex is required for EP300 recruitment. Single‐ or dual‐knockdown CTCF and PACERR THP‐1 cells were constructed using lentivirus. Knockdown of CTCF and PACERR did not affect the overall expression of EP300 (Figure S18F). However, ChIP‐qPCR assays showed that both CTCF and PACERR are indispensable for the binding of EP300 to the promoter region of PTGS2 (Figure 7F). To understand the mechanism of PTGS2 by EP300, we conducted a stable knockdown of EP300 expression in THP‐1 cells (Figure 7G) and performed chromatin immunoprecipitation using anti‐H3K27ac antibodies. ChIP‐qPCR assays show that the enrichment of EP300 was significantly reduced at the PTGS2 promoter in the shEP300‐TAMs group (Figure S19). And the protein levels of PTGS2 were indeed down‐regulated after knockdown EP300 in THP‐1‐derived TAMs (Figure 7H). In addition, we observed that the RNA level of PACERR was decreased after EP300 was successfully knocked down in TAMs to confirm the regulation response of EP300 to PACERR (Figure 7J). Taken together, these findings indicate that CTCF/PACERR complex is required for recruitment of EP300 to the promoter region of PTGS2, resulting in EP300‐induced histone acetylation and chromatin accessibility.

**FIGURE 8:**
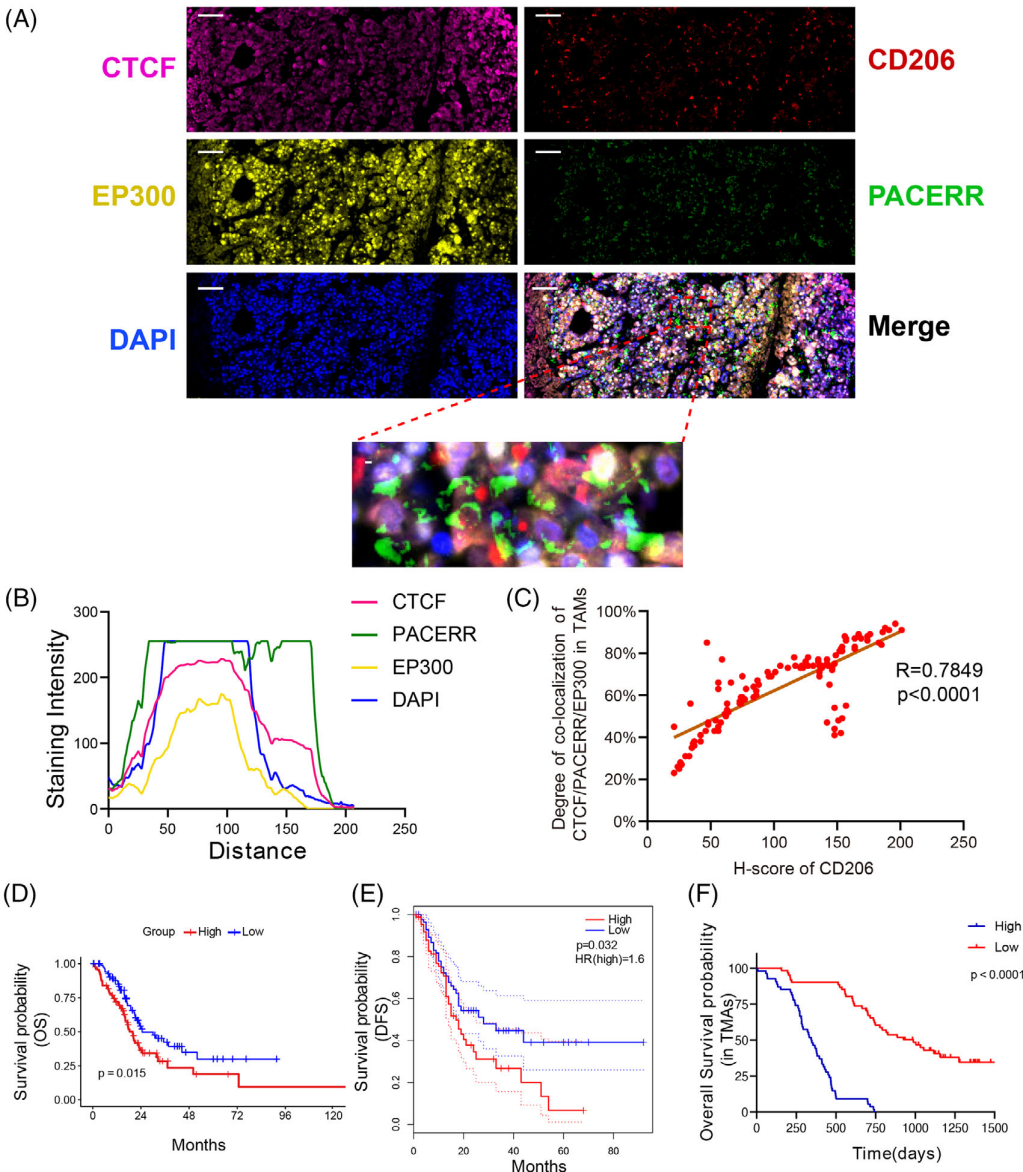
The relationship among CCCTC‐binding factor (CTCF), PTGS2 antisense NF‐κB1 complex‐mediated expression regulator RNA (PACERR) and E1A binding protein p300 (EP300) in tumour‐associated macrophages (TAMs) and prediction values of CTCF, PACERR and EP300 in pancreatic ductal adenocarcinoma (PDAC). (A) The immunofluorescence and fluorescence in situ hybridisation (FISH) analysis of CTCF (pink), EP300 (yellow), CD206 (red), PACERR (green) and DAPI (blue) in PDAC tissues. Scar bar: 20μm. (B) Staining intensity of CTCF, PACERR, EP300 and DAPI on the immunofluorescence from TAMs of 110 PDAC patients. Pink represents CTCF. Green represents PACERR. Yellow represents EP300. Blue represents DAPI. (C) The Spearman correlation between the expression of CD206 and the degree of co‐localisation of CTCF, PACERR and EP300. The rates of the triple‐positive (CTCF^+^‐PACERR^+^‐EP300^+^) in TAMs were used to evaluate the degree of colocalisation of CTCF, PACERR and EP300. (D) and (E) PAAD patients from TCGA were divided into high group and low group based on the median expression of the multi‐signature of CTCF, PACERR and EP300. Kaplan‐Meier curves analysis of the overall survival (OS) (D) and disease‐free survival (E) between two groups. (F) 110 PDAC patients were divided into high group and low group based on the median of rates of triple‐positive cells in TAMs (the percentage of CTCF^+^‐PACERR^+^‐EP300^+^ TAMs number in total CD206 positive cells number). Kaplan‐Meier curves analysis of the overall survival (OS) between two groups

To further verify the relationship among CTCF, PACERR and EP300 in TAMs, we performed four‐color IF (IF) staining of CTCF, EP300 and CD206 and FISH staining of PACERR on one section from TMAs of 110 PDAC patients (Figure 8A). We found that there was a high degree of co‐localisation of CTCF, PACERR and EP300 in CD206^+^ TAMs and the expression of CD206 and the degree of co‐localisation of CTCF, PACERR and EP300 were positively related (Figure 8B,C). To explore the prediction values of CTCF, PACERR and EP300 in PDAC, we performed survival analysis based on the median expression status of a multi‐gene signature of CTCF, PACERR and EP300 for PAAD patients in The Cancer Genome Atlas (TCGA) database and plot Kaplan‐Meier curves, revealing that a high expression of the multi‐signature of CTCF, PACERR and EP300 was significantly associated with poor OS (Figure 8D) and disease‐free survival (Figure 8E). Consistent with this, the 110 PDAC patients were divided into high group and low group based on the median of rates of triple‐positive cells in TAMs (the percentage of CTCF^+^‐PACERR^+^‐EP300^+^ TAMs number in total CD206 positive cells number), and Kaplan‐Meier curves were also verified that the multi‐signature of CTCF, PACERR and EP300 was a potential poor prognostic marker in PDAC (Figure 8F).

## 4 DISCUSSION

**FIGURE 9:**
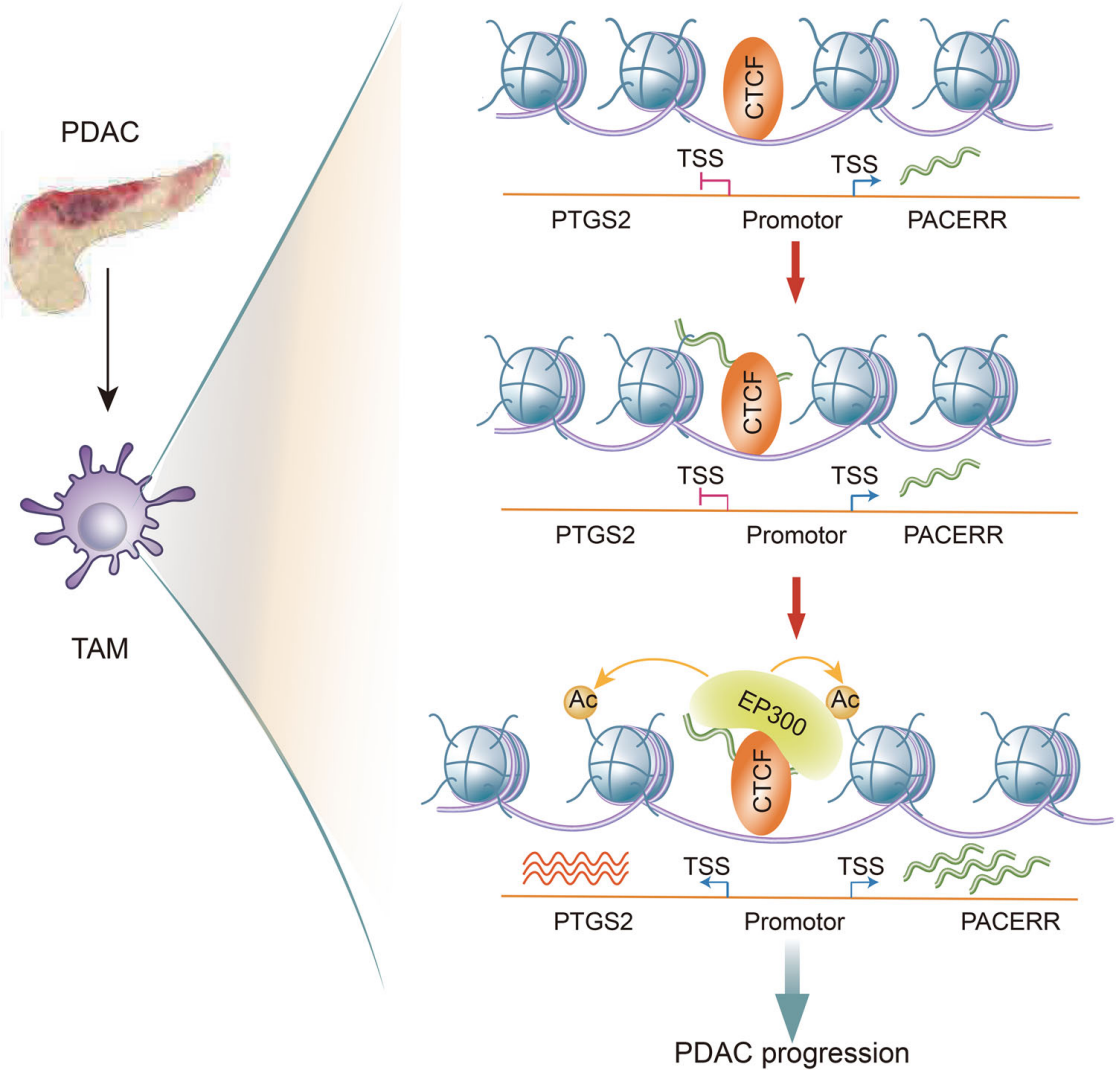
A model of CCCTC‐binding factor (CTCF)‐controlled prostaglandin‐endoperoxide synthase 2 (PTGS2) expression in tumour‐associated macrophages (TAMs) in the pancreatic ductal adenocarcinoma (PDAC) tumour microenvironment. CTCF forms a complex with the *cis*‐regulatory long noncoding RNA‐PTGS2 antisense NF‐κB1 complex‐mediated expression regulator RNA (LncRNA‐PACERR) in the PTGS2 promoter region and recruits E1A binding protein p300 (EP300), to enhance chromatin accessibility and promote the transcription of PTGS2, thereby inducing M2 polarization and pro‐tumour functions of TAMs

TAMs are an extremely plastic population that acquire distinct phenotypes and functions in response to a variety of environmental cues,^15^, ^16^ and epigenetic modulation plays an important role in macrophage reprogramming in the tumour microenvironment.^15^, ^18^ It has been reported that DNA methylation, histone modification and microRNA regulate the differentiation and function of TAMs towards either anti‐tumour M1 macrophages or pro‐tumour M2 macrophages.^15^, ^18^ Here, we discovered a novel mechanism of epigenetic modulation, that results in polarization of TAMs towards pro‐tumour M2 phenotype: CTCF binds to the overlapped promoter region of PACERR and PTGS2, leading to the transcription of PACERR. The newly transcribed LncRNA PACERR interacts directly with CTCF, forming the CTCF/PACERR complex to recruit HAT EP300, resulting in increased chromatin accessibility and transcriptional activation of PTGS2, the critical driver of M2 polarization and pro‐tumour functions in TAMs (Figure 9).

Interestingly, we found that the expression of M2 markers was diminished and classical M1 markers were not changed after CTCF knockdown (Figure 2A) and PACERR knockdown (Figure 5A) in THP‐1‐derived TAMs. However, the number of CD68^+^ cells which should contain M1 and M2 macrophages were no difference in metastatic foci in the livers of mice between the treatment group and control group (Figures S4D and S5D). The Hallmarks of the inflammatory response, interferon‐α response, interferon‐γ response and TNF‐α signalling via NF‐κB were enriched after CTCF knockdown in TAMs by Gene Set Enrichment Analysis (Figure S20A–D). Therefore, we asked whether CTCF and PACERR may promote the polarization of non‐classical M1 macrophages to M2 macrophages. The expression of non‐classical M1 markers CD40, CD74 and CD70 were increased after CTCF or PACERR knockdown by qPCR analysis. (Figure S21A,B) Flow cytometric analyses confirmed that a decrease of CTCF or PACERR expression in TAMs resulted in a significantly increased percentage of CD40^+^, CD74^+^ and CD70^+^ cells (non‐classical M1 macrophages). (Figure S22A–F). Thus, CTCF and PACERR may facilitate the metastasis of PDAC via the non‐classical M1–M2 polarization.

CTCF is a DNA binding factor with diverse regulatory functions. CTCF could participate in the regulation of high‐dimensional chromatin structure, enhancer/promoter insulation, direct transcriptional regulation, RNA splicing, and RNA binding.^33^, ^34^ Interestingly, our study reflected several aspects of CTCF function, including promoting the formation of transcription initiation complexes, affecting chromatin accessibility, and interacting with LncRNA PACERR. Of note, most of the previous studies on the RNA binding activity of CTCF demonstrated the involvement of the RNA molecules in CTCF‐mediated chromatin remodelling,^43^, ^44^ while our study revealed a novel mechanism that CTCF regulates the transcription of LncRNA‐PACERR, which participates in the *cis*‐regulation of downstream genes via directly interacting with CTCF. Consistent with previous knowledge that CTCF could regulate gene transcription by interacting with HAT or histone deacetylase, our results indicated that the CTCF/PACERR complex positively regulates PTGS2 transcription via recruiting the HAT EP300, that transfers acetyl groups to histones to enhance chromatin accessibility. Notably, THP‐1 cells which were cultured from the blood of a boy with acute monocytic leukaemia (reference) were not normal macrophages. In future studies, we will further address the question of whether PTGS2 expression in TAMs is also regulated by CTCF‐mediated high‐dimensional chromatin remodelling and long‐range enhancer‐promoter interactions. We will apply HiC, 3C and other state‐of‐art technologies to further investigate these questions.

PTGS2 (also known as COX‐2) has been well recognised as an important driver of M2 polarization and pro‐tumour functions of TAMs.^36^, ^49^ In Apc^Min/+^ mouse model of intestinal tumorigenesis, COX‐2 overexpression in TAMs resulted in enhanced tumour growth, increased dysplasia, and submucosal tumour invasion.^50^ Consistently, TAMs were found to promote the survival, EMT and metastatic potential of breast cancer cells via COX‐2‐mediated intercellular communication.^41^ It has also been reported that the COX‐2/PGE2 axis induces immunosuppressive functions in myeloid cells, and COX‐2/PGE2 blockade could reverse the immunosuppression, activate anti‐tumour immunity, and ultimately cause tumour regression.^51^, ^52^ Therefore, PTGS2 (COX‐2) expression in TAMs could be a promising therapeutic target to re‐educate the TAMs to abrogate their pro‐tumour activity and re‐activate immunosurveillance.

However, due to the significant off‐target side effects of currently available agents for PTGS2 (COX‐2) blockade,^53^ modulating COX‐2 transcription could be an attractive alternative approach. Our study revealed a novel mechanism of regulation of PTGS2 expression in TAMs, providing insights for the design of therapeutic approaches interfering with PTGS2 expression. Indeed, PACERR expression in TAMs could be a promising alternative target, as LncRNAs could be successfully depleted by siRNAs or antisense oligonucleotides.^54^, ^55^ In addition, it is also feasible to interfere with the binding of CTCF or PACERR with PTGS2 locus. Newly‐developed nanoparticles with enhanced tumour penetration could overcome the biological barriers of the heavy desmoplastic reaction in the PDAC tumour microenvironment,^56^ and these nanoparticles targeting PTGS2 (COX‐2) expression in CD206^+^ TAMs would be predicted to have therapeutic benefits for PDAC patients.

## 5 CONCLUSIONS

We demonstrated that CTCF‐transcribed LncRNA PACERR directly interacts with CTCF, and the CTCF/PACERR complex recruits the acetyltransferase EP300 to increase the chromatic accessibility of PTGS2 locus, resulting in upregulated PTGS2 expression, thereby promoting the M2 polarization and pro‐tumour functions of TAMs.

## CONFLICT OF INTEREST

The authors declare to have no conflict of interest.

## Supporting information



Supporting Information

Supporting Information

Supporting Information

Supporting Information

Supporting Information
