# Supplementary material for: The CTCF/LncRNA‐PACERR complex recruits E1A binding protein p300 to induce pro‐tumour macrophages in pancreatic ductal adenocarcinoma via directly regulating PTGS2 expression
Source: Clin Transl Med. 2022 Feb 20;12(2):e654. doi: 10.1002/ctm2.654 (PMC8858628; doi:10.1002/ctm2.654)
Supplement: Supplementary file 1 — Supporting Information [file CTM2-12-e654-s003.pdf]

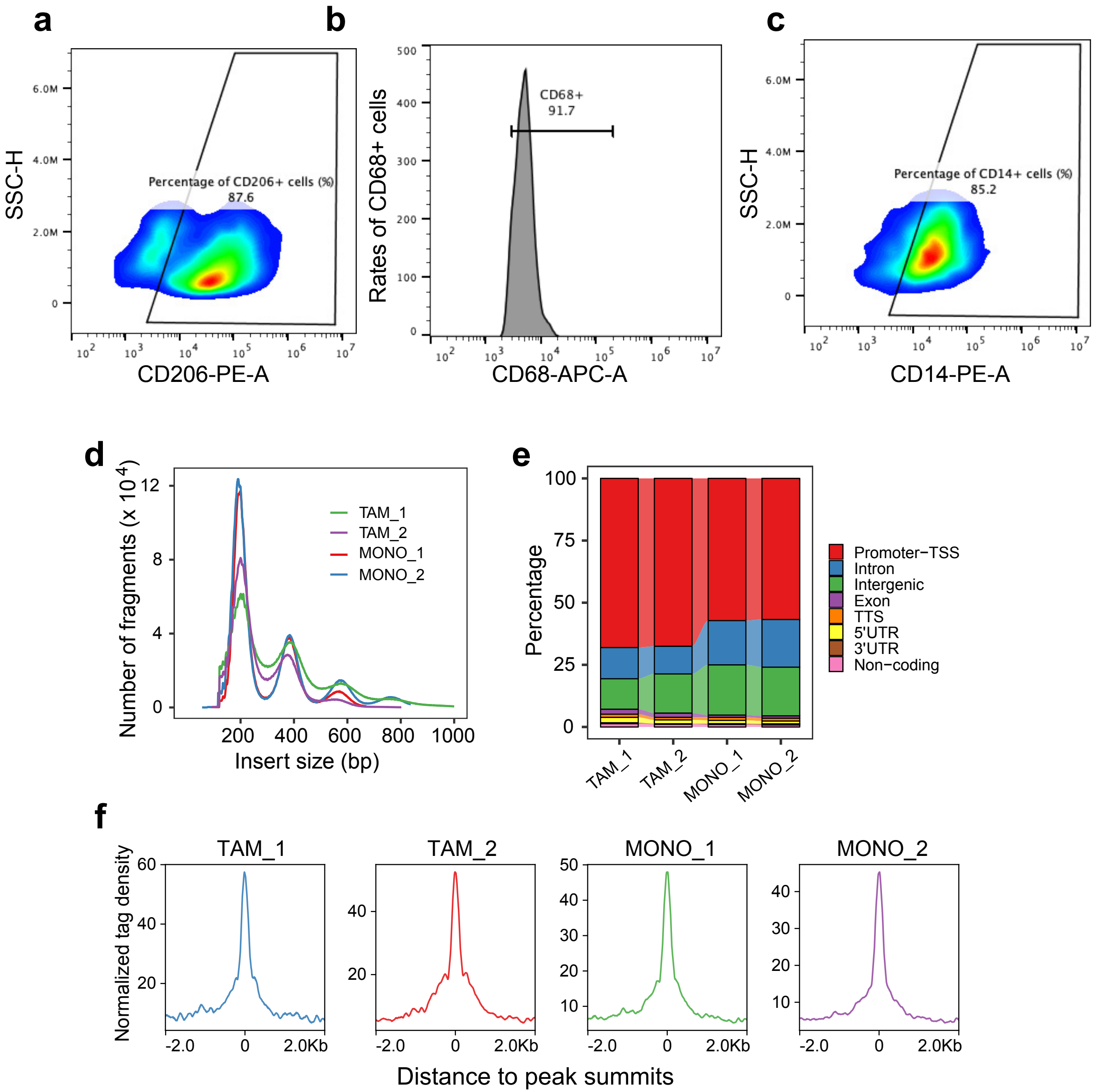

a
SSC-H
6.0M
4.0M
2.0M
0
Percentage of CD206+ cells (%)
87.6
CD206-PE-A
b
Rates of CD68+ cells
500
400
300
200
100
0
CD68+
91.7
CD68-APC-A
c
SSC-H
6.0M
4.0M
2.0M
0
Percentage of CD14+ cells (%)
85.2
CD14-PE-A
d
Number of fragments (x 10^4)
TAM_1
TAM_2
MONO_1
MONO_2
200 400 600 800 1000
Insert size (bp)
e
Percentage
100
75
50
25
0
Promoter−TSS
Intron
Intergenic
Exon
TTS
5'UTR
3'UTR
Non−coding
TAM_1
TAM_2
MONO_1
MONO_2
f
Normalized tag density
TAM_1
TAM_2
MONO_1
MONO_2
-2.0
0
2.0Kb
Distance to peak summits

Figure S1

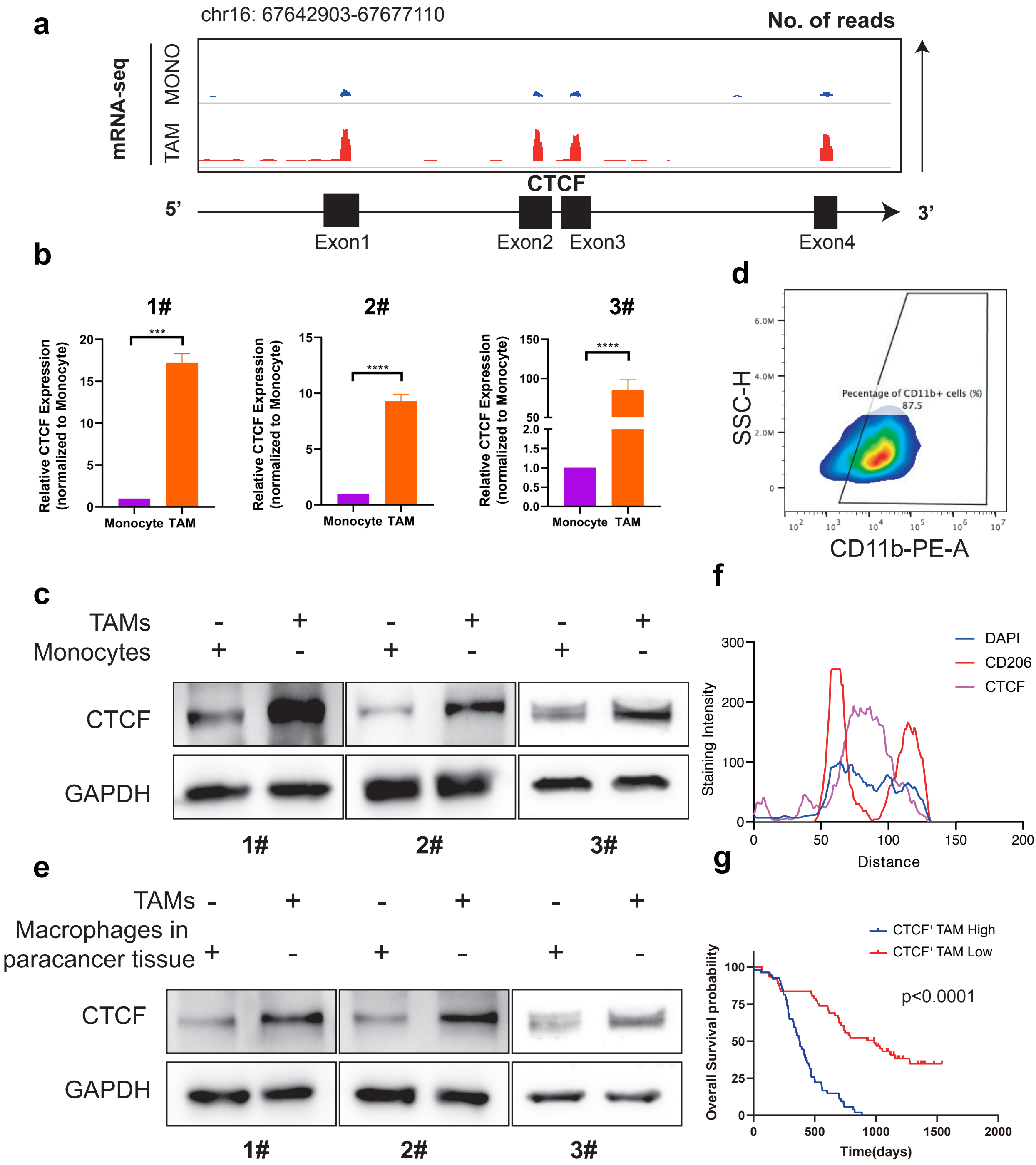
a
chr16: 67642903-67677110
No. of reads
mRNA-seq
MONO
TAM
CTCF
5'
3'
Exon1
Exon2
Exon3
Exon4
b
1#
2#
3#
Relative CTCF Expression (normalized to Monocyte)
***
****
****
Monocyte TAM
d
SSC-H
Pecentage of CD11b+ cells (%) 87.5
CD11b-PE-A
c
TAMs
Monocytes
CTCF
GAPDH
1#
2#
3#
f
DAPI
CD206
CTCF
Staining Intensity
Distance
e
TAMs
Macrophages in paracancer tissue
CTCF
GAPDH
1#
2#
3#
g
CTCF⁺ TAM High
CTCF⁺ TAM Low
p<0.0001
Overall Survival probability
Time(days)

Figure S2

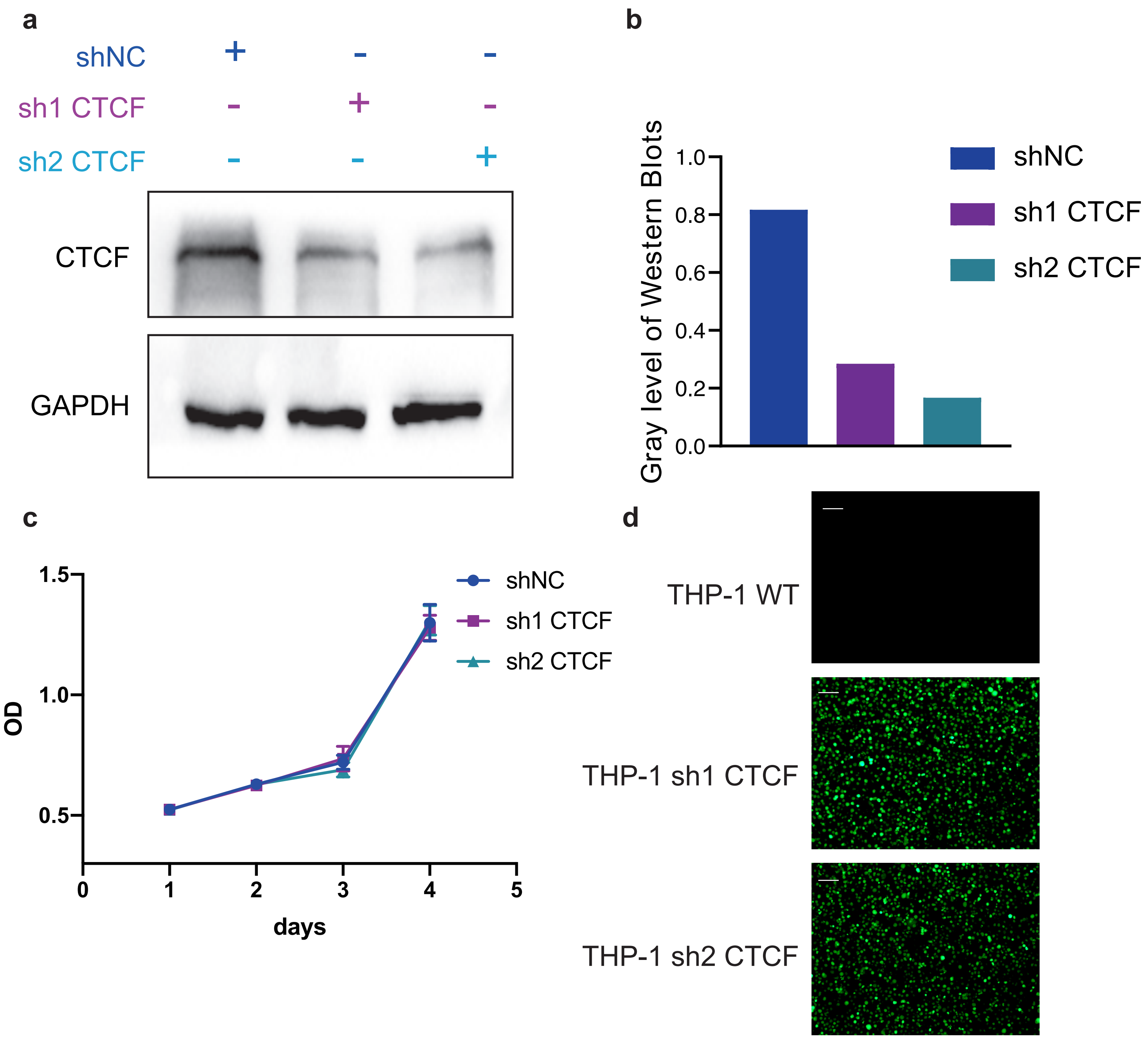
a
shNC + - -
sh1 CTCF - + -
sh2 CTCF - - +
CTCF
GAPDH
b
Gray level of Western Blots
1.0
0.8
0.6
0.4
0.2
0.0
shNC
sh1 CTCF
sh2 CTCF
c
OD
1.5
1.0
0.5
0
1
2
3
4
5
days
shNC
sh1 CTCF
sh2 CTCF
d
THP-1 WT
THP-1 sh1 CTCF
THP-1 sh2 CTCF

Figure S3

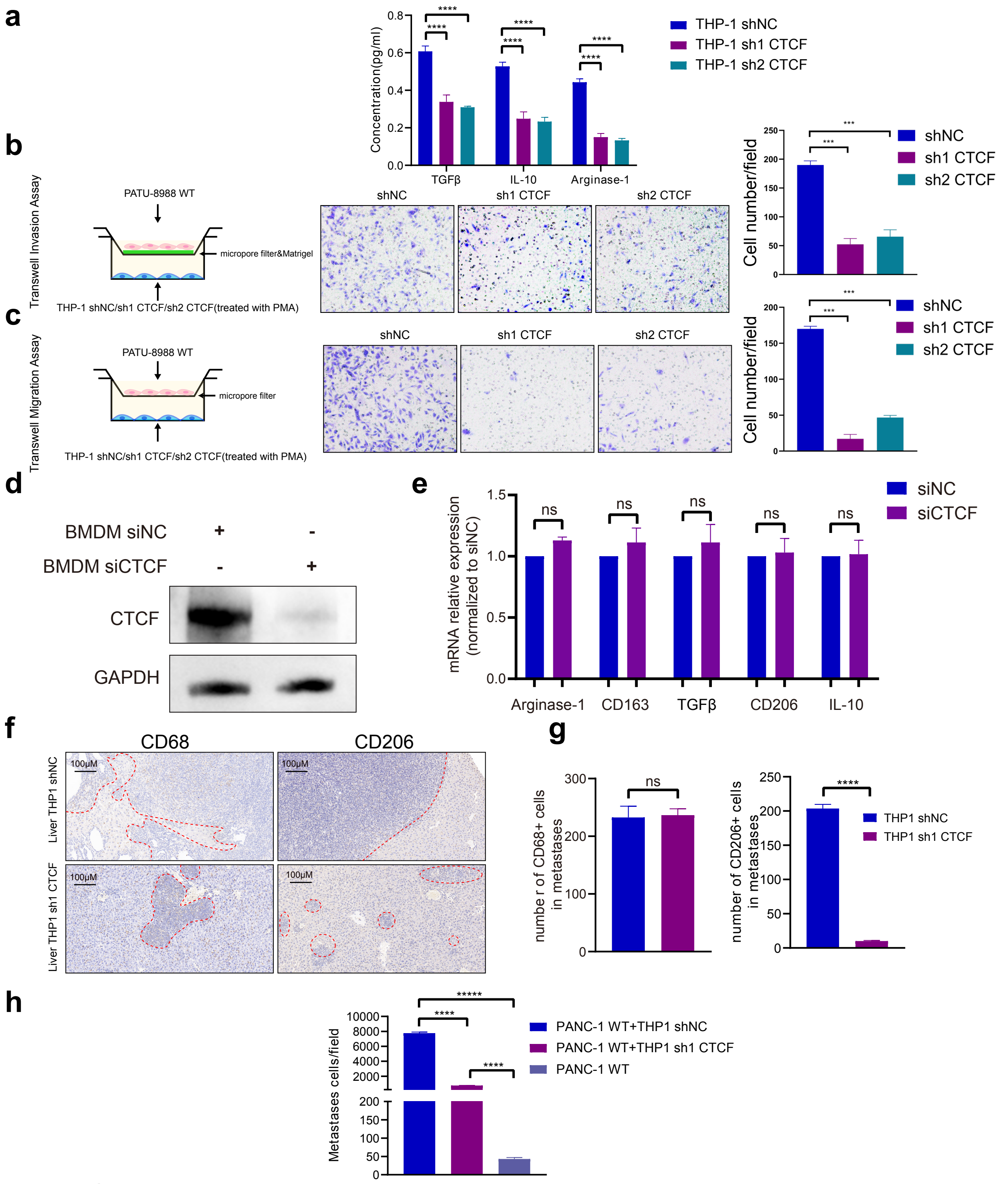
a
Concentration(pg/ml)
TGFβ
IL-10
Arginase-1
THP-1 shNC
THP-1 sh1 CTCF
THP-1 sh2 CTCF
b
Transwell Invasion Assay
PATU-8988 WT
micropore filter&Matrigel
THP-1 shNC/sh1 CTCF/sh2 CTCF(treated with PMA)
shNC
sh1 CTCF
sh2 CTCF
Cell number/field
c
Transwell Migration Assay
PATU-8988 WT
micropore filter
THP-1 shNC/sh1 CTCF/sh2 CTCF(treated with PMA)
d
BMDM siNC
BMDM siCTCF
CTCF
GAPDH
e
mRNA relative expression
(normalized to siNC)
Arginase-1
CD163
TGFβ
CD206
IL-10
siNC
siCTCF
f
CD68
CD206
Liver THP1 shNC
Liver THP1 sh1 CTCF
100μM
g
numbe r of CD68+ cells
in metastases
number of CD206+ cells
in metastases
THP1 shNC
THP1 sh1 CTCF
h
Metastases cells/field
PANC-1 WT+THP1 shNC
PANC-1 WT+THP1 sh1 CTCF
PANC-1 WT

Figure S4

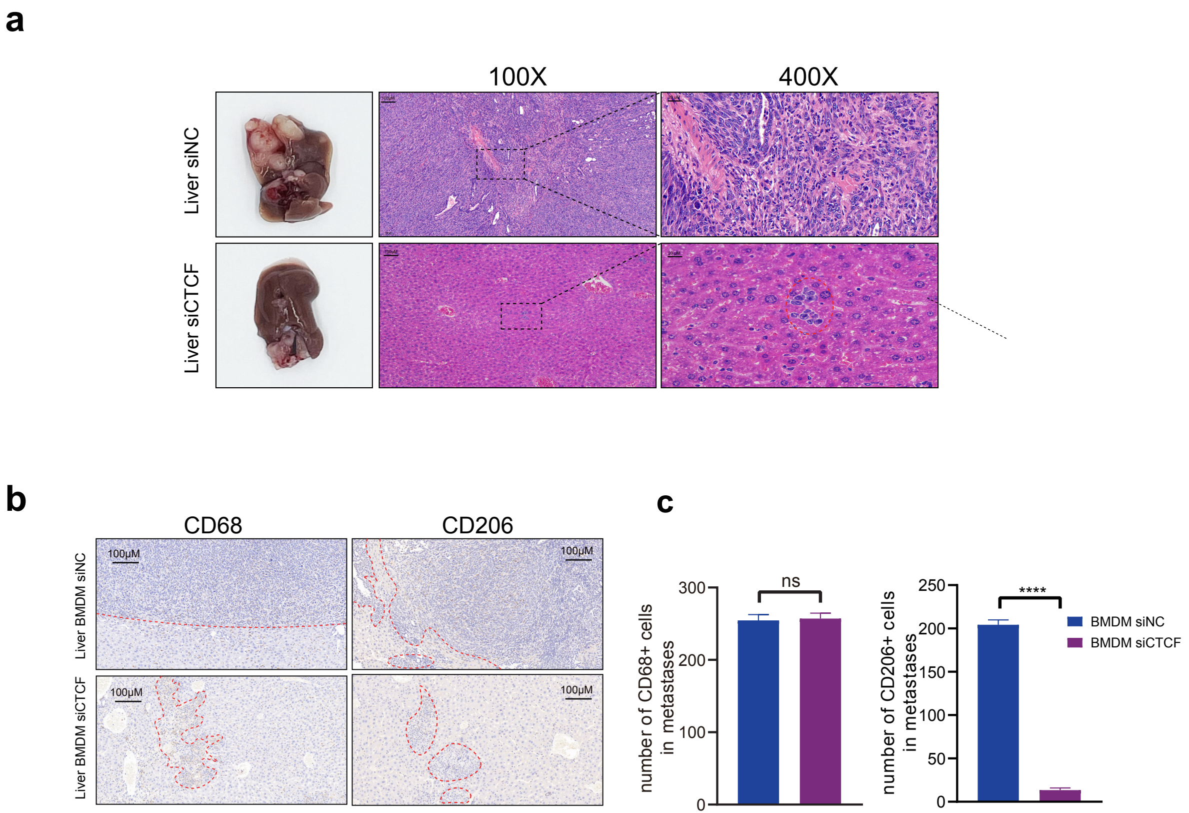
a
100X
400X
Liver siNC
Liver siCTCF
b
CD68
CD206
Liver BMDM siNC
Liver BMDM siCTCF
100μM
c
ns
****
number of CD68+ cells in metastases
number of CD206+ cells in metastases
300
200
100
0
250
150
50
BMDM siNC
BMDM siCTCF

Figure S5

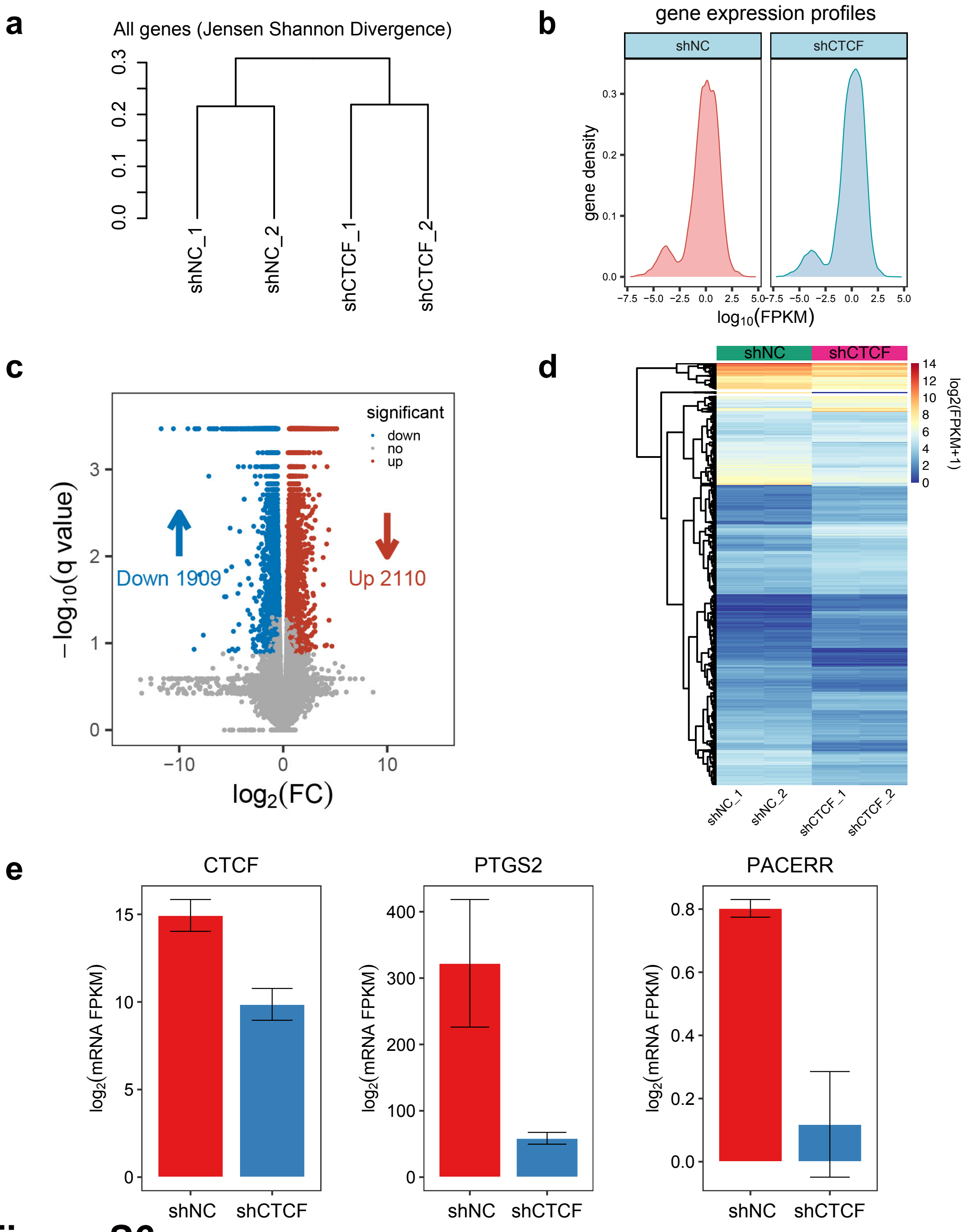
a
All genes (Jensen Shannon Divergence)
0.0
0.1
0.2
0.3
shNC_1
shNC_2
shCTCF_1
shCTCF_2
b
gene expression profiles
shNC
shCTCF
gene density
log10(FPKM)
c
significant
down
no
up
Down 1909
Up 2110
−log10(q value)
log2(FC)
d
shNC
shCTCF
log2(FPKM+1)
shNC_1
shNC_2
shCTCF_1
shCTCF_2
e
CTCF
PTGS2
PACERR
log2(mRNA FPKM)
shNC
shCTCF

Figure S6

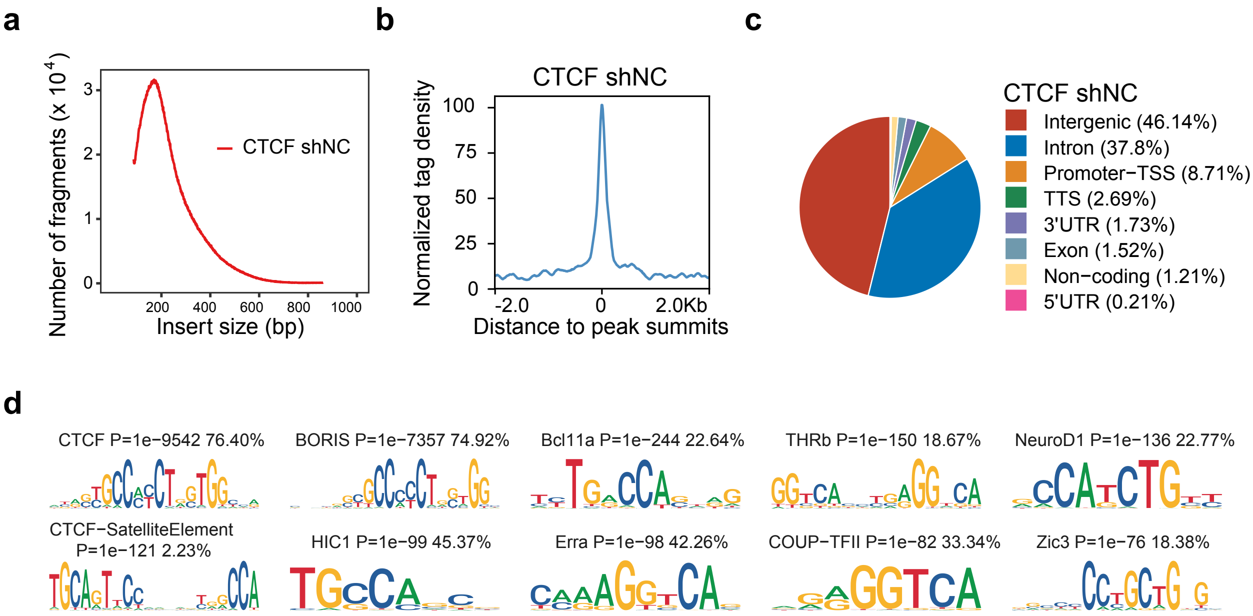
a
Number of fragments (x 10^4)
3
2
1
0
200
400
600
800
1000
Insert size (bp)
CTCF shNC
b
CTCF shNC
Normalized tag density
100
75
50
25
0
-2.0
0
2.0Kb
Distance to peak summits
c
CTCF shNC
Intergenic (46.14%)
Intron (37.8%)
Promoter−TSS (8.71%)
TTS (2.69%)
3'UTR (1.73%)
Exon (1.52%)
Non−coding (1.21%)
5'UTR (0.21%)
d
CTCF P=1e−9542 76.40%
BORIS P=1e−7357 74.92%
Bcl11a P=1e−244 22.64%
THRb P=1e−150 18.67%
NeuroD1 P=1e−136 22.77%
CTCF−SatelliteElement P=1e−121 2.23%
HIC1 P=1e−99 45.37%
Erra P=1e−98 42.26%
COUP−TFII P=1e−82 33.34%
Zic3 P=1e−76 18.38%

Figure S7

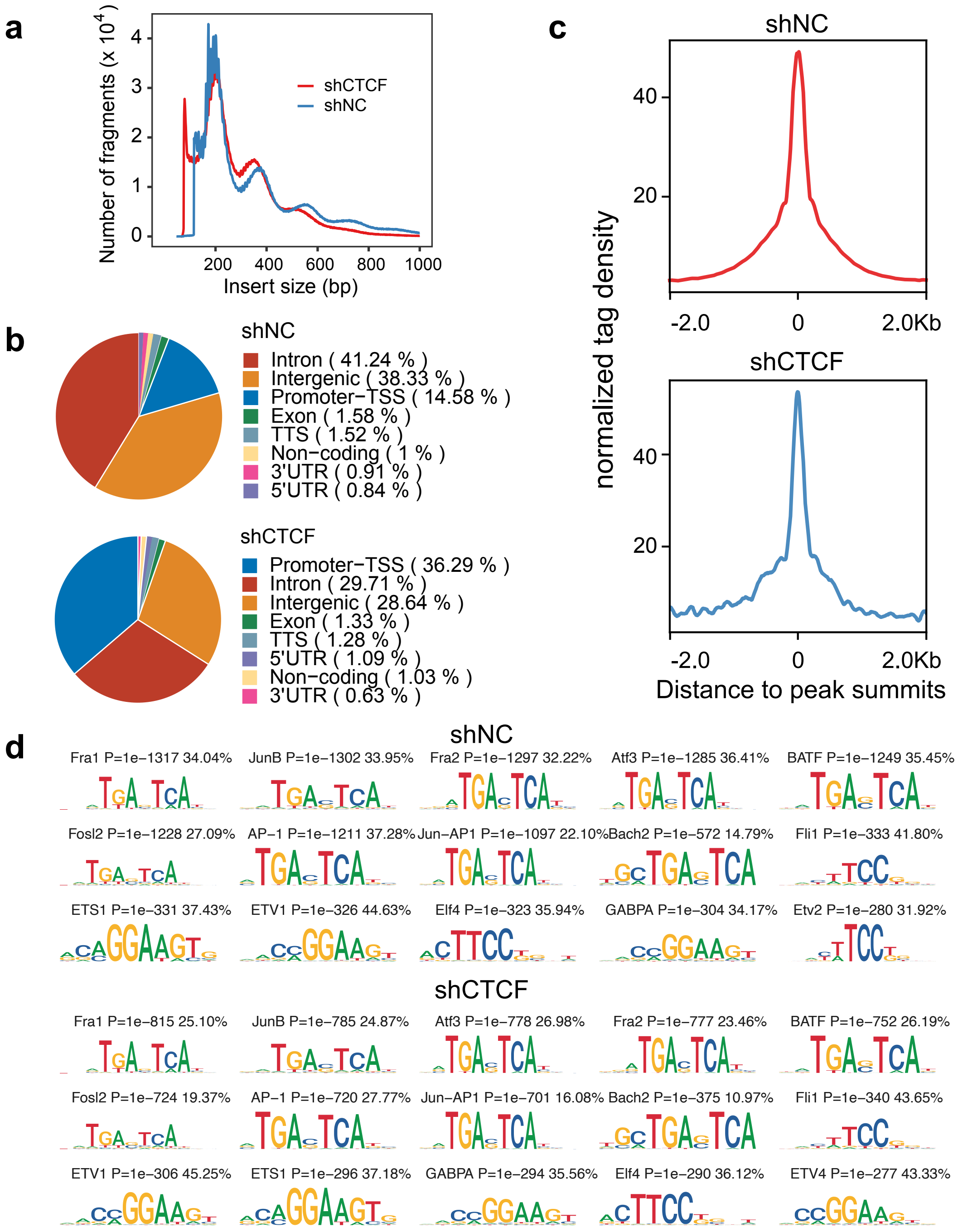
a
Number of fragments (x 10^4)
Insert size (bp)
shCTCF
shNC
b
shNC
Intron (41.24 %)
Intergenic (38.33 %)
Promoter−TSS (14.58 %)
Exon (1.58 %)
TTS (1.52 %)
Non−coding (1 %)
3'UTR (0.91 %)
5'UTR (0.84 %)
shCTCF
Promoter−TSS (36.29 %)
Intron (29.71 %)
Intergenic (28.64 %)
Exon (1.33 %)
TTS (1.28 %)
5'UTR (1.09 %)
Non−coding (1.03 %)
3'UTR (0.63 %)
c
shNC
shCTCF
normalized tag density
Distance to peak summits
d
shNC
Fra1 P=1e−1317 34.04%
JunB P=1e−1302 33.95%
Fra2 P=1e−1297 32.22%
Atf3 P=1e−1285 36.41%
BATF P=1e−1249 35.45%
Fosl2 P=1e−1228 27.09%
AP−1 P=1e−1211 37.28%
Jun−AP1 P=1e−1097 22.10%
Bach2 P=1e−572 14.79%
Fli1 P=1e−333 41.80%
ETS1 P=1e−331 37.43%
ETV1 P=1e−326 44.63%
Elf4 P=1e−323 35.94%
GABPA P=1e−304 34.17%
Etv2 P=1e−280 31.92%
shCTCF
Fra1 P=1e−815 25.10%
JunB P=1e−785 24.87%
Atf3 P=1e−778 26.98%
Fra2 P=1e−777 23.46%
BATF P=1e−752 26.19%
Fosl2 P=1e−724 19.37%
AP−1 P=1e−720 27.77%
Jun−AP1 P=1e−701 16.08%
Bach2 P=1e−375 10.97%
Fli1 P=1e−340 43.65%
ETV1 P=1e−306 45.25%
ETS1 P=1e−296 37.18%
GABPA P=1e−294 35.56%
Elf4 P=1e−290 36.12%
ETV4 P=1e−277 43.33%

Figure S8

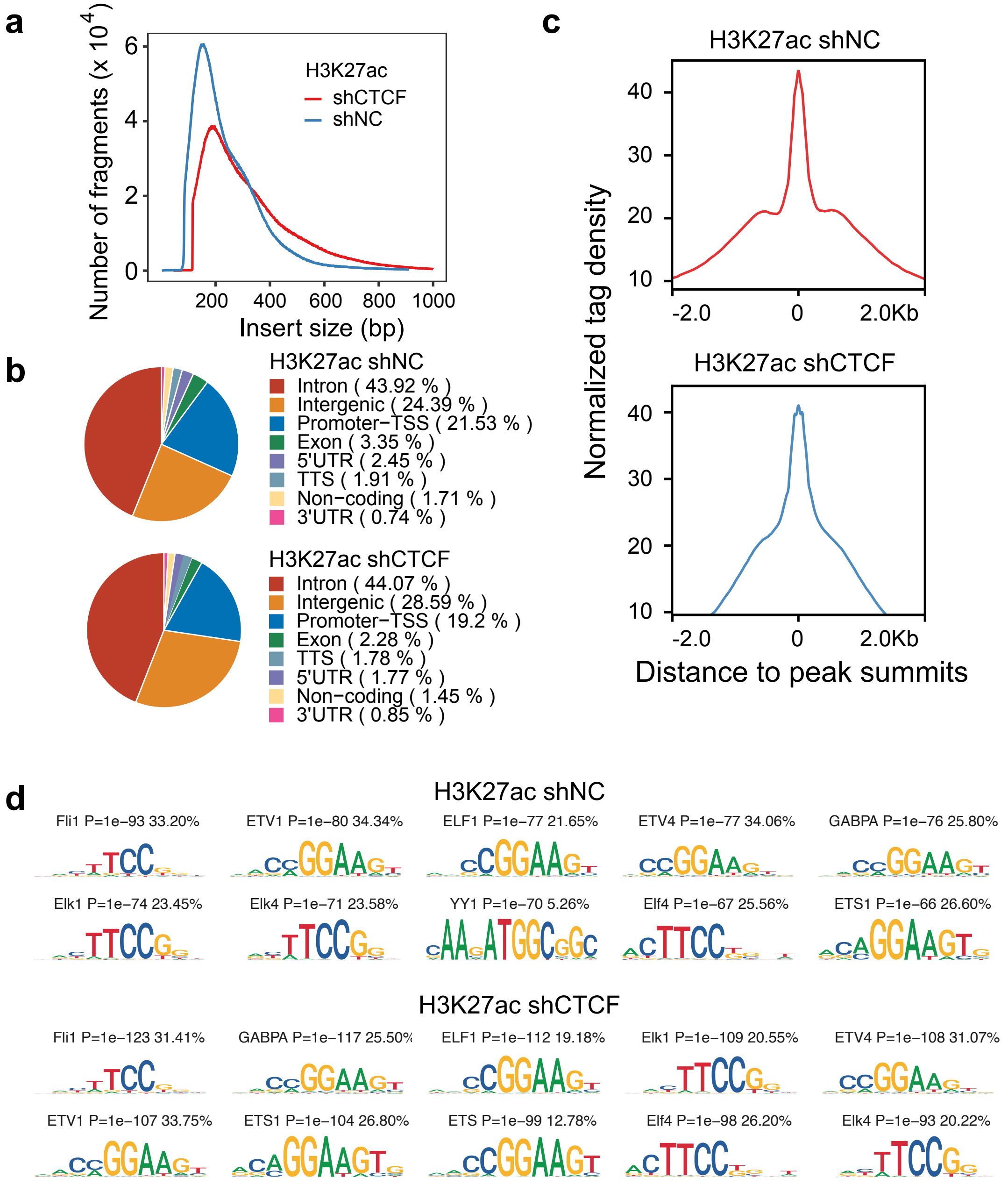
a
H3K27ac
shCTCF
shNC
Number of fragments (x 10^4)
Insert size (bp)
b
H3K27ac shNC
Intron (43.92 %)
Intergenic (24.39 %)
Promoter−TSS (21.53 %)
Exon (3.35 %)
5'UTR (2.45 %)
TTS (1.91 %)
Non−coding (1.71 %)
3'UTR (0.74 %)
H3K27ac shCTCF
Intron (44.07 %)
Intergenic (28.59 %)
Promoter−TSS (19.2 %)
Exon (2.28 %)
TTS (1.78 %)
5'UTR (1.77 %)
Non−coding (1.45 %)
3'UTR (0.85 %)
c
H3K27ac shNC
H3K27ac shCTCF
Normalized tag density
Distance to peak summits
d
H3K27ac shNC
Fli1 P=1e−93 33.20%
ETV1 P=1e−80 34.34%
ELF1 P=1e−77 21.65%
ETV4 P=1e−77 34.06%
GABPA P=1e−76 25.80%
Elk1 P=1e−74 23.45%
Elk4 P=1e−71 23.58%
YY1 P=1e−70 5.26%
Elf4 P=1e−67 25.56%
ETS1 P=1e−66 26.60%
H3K27ac shCTCF
Fli1 P=1e−123 31.41%
GABPA P=1e−117 25.50%
ELF1 P=1e−112 19.18%
Elk1 P=1e−109 20.55%
ETV4 P=1e−108 31.07%
ETV1 P=1e−107 33.75%
ETS1 P=1e−104 26.80%
ETS P=1e−99 12.78%
Elf4 P=1e−98 26.20%
Elk4 P=1e−93 20.22%

Figure S9

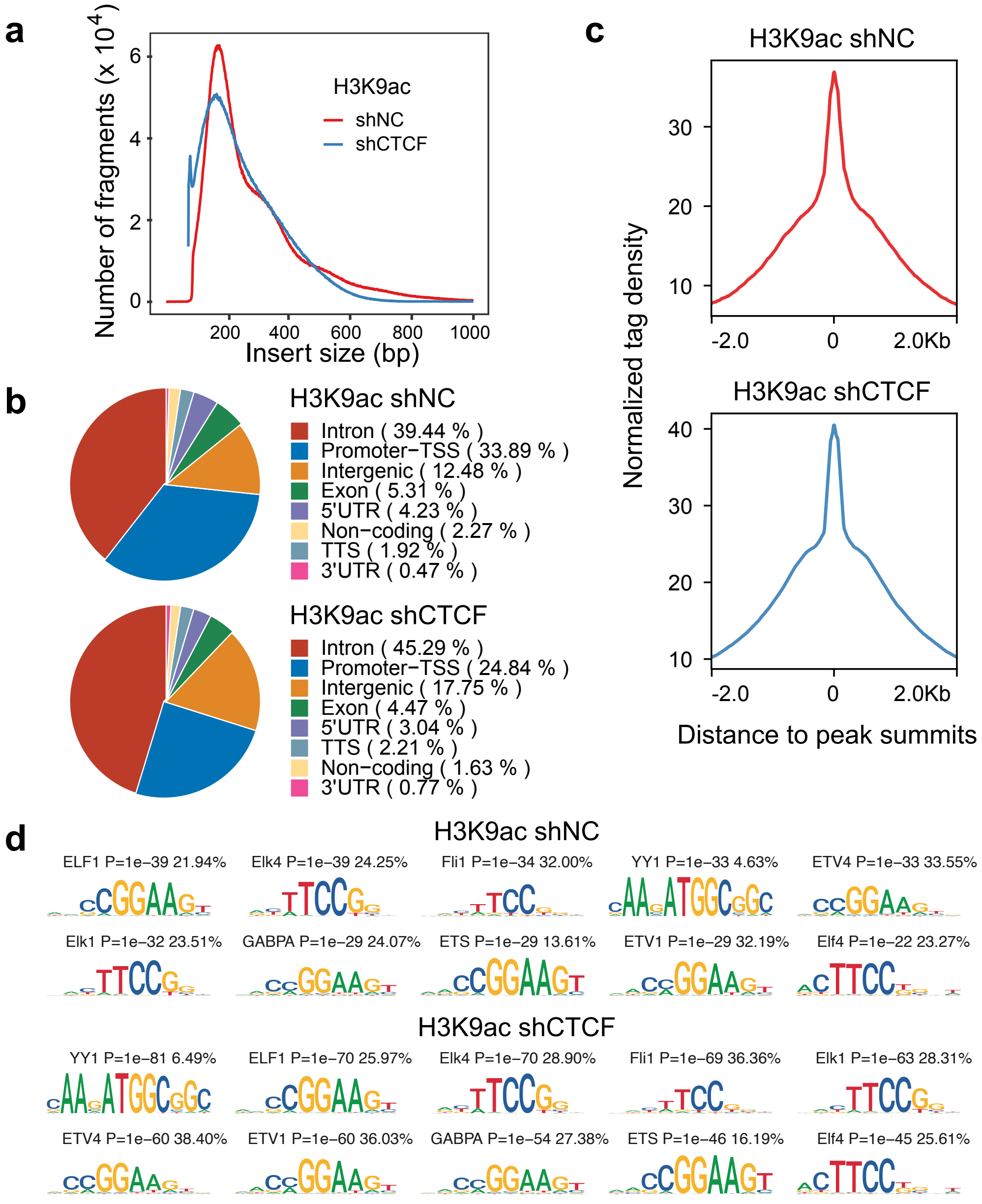
a
H3K9ac
shNC
shCTCF
Number of fragments (x 10^4)
0
2
4
6
200
400
600
800
1000
Insert size (bp)
b
H3K9ac shNC
Intron (39.44 %)
Promoter−TSS (33.89 %)
Intergenic (12.48 %)
Exon (5.31 %)
5'UTR (4.23 %)
Non−coding (2.27 %)
TTS (1.92 %)
3'UTR (0.47 %)
H3K9ac shCTCF
Intron (45.29 %)
Promoter−TSS (24.84 %)
Intergenic (17.75 %)
Exon (4.47 %)
5'UTR (3.04 %)
TTS (2.21 %)
Non−coding (1.63 %)
3'UTR (0.77 %)
c
H3K9ac shNC
H3K9ac shCTCF
Normalized tag density
10
20
30
40
-2.0
0
2.0Kb
Distance to peak summits
d
H3K9ac shNC
ELF1 P=1e−39 21.94%
Elk4 P=1e−39 24.25%
Fli1 P=1e−34 32.00%
YY1 P=1e−33 4.63%
ETV4 P=1e−33 33.55%
Elk1 P=1e−32 23.51%
GABPA P=1e−29 24.07%
ETS P=1e−29 13.61%
ETV1 P=1e−29 32.19%
Elf4 P=1e−22 23.27%
H3K9ac shCTCF
YY1 P=1e−81 6.49%
ELF1 P=1e−70 25.97%
Elk4 P=1e−70 28.90%
Fli1 P=1e−69 36.36%
Elk1 P=1e−63 28.31%
ETV4 P=1e−60 38.40%
ETV1 P=1e−60 36.03%
GABPA P=1e−54 27.38%
ETS P=1e−46 16.19%
Elf4 P=1e−45 25.61%

Figure S10

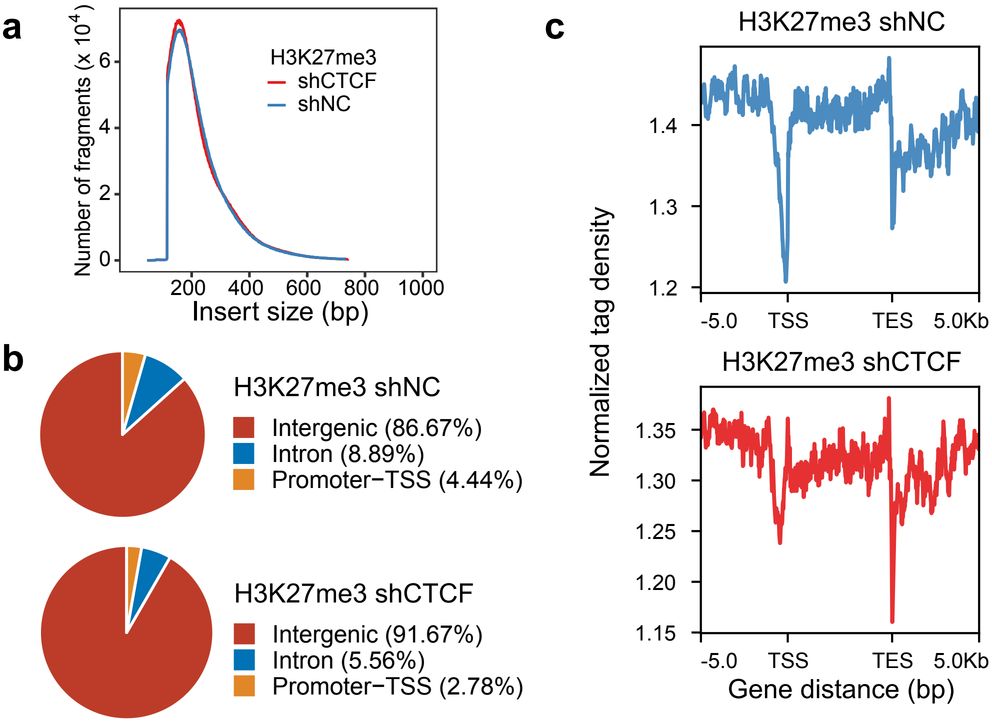
a
Number of fragments (x 10^4)
0
2
4
6
200
400
600
800
1000
Insert size (bp)
H3K27me3
shCTCF
shNC
b
H3K27me3 shNC
Intergenic (86.67%)
Intron (8.89%)
Promoter−TSS (4.44%)
H3K27me3 shCTCF
Intergenic (91.67%)
Intron (5.56%)
Promoter−TSS (2.78%)
c
H3K27me3 shNC
1.4
1.3
1.2
-5.0
TSS
TES
5.0Kb
H3K27me3 shCTCF
1.35
1.30
1.25
1.20
1.15
-5.0
TSS
TES
5.0Kb
Normalized tag density
Gene distance (bp)

Figure S11

a

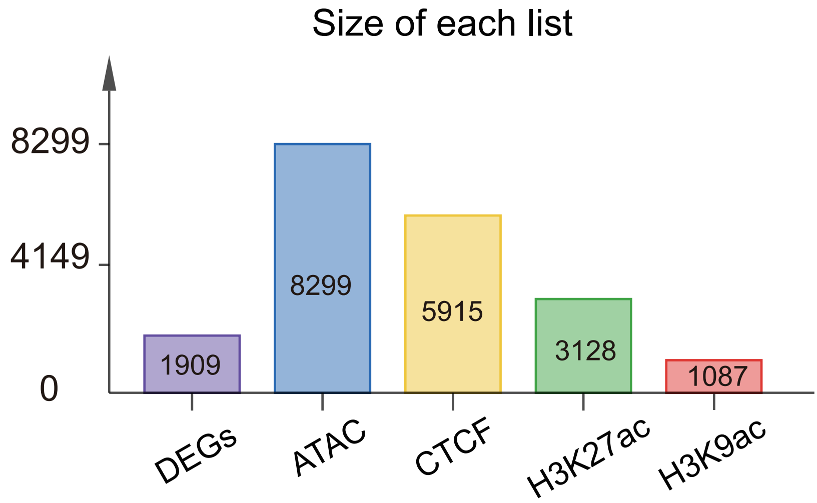

b

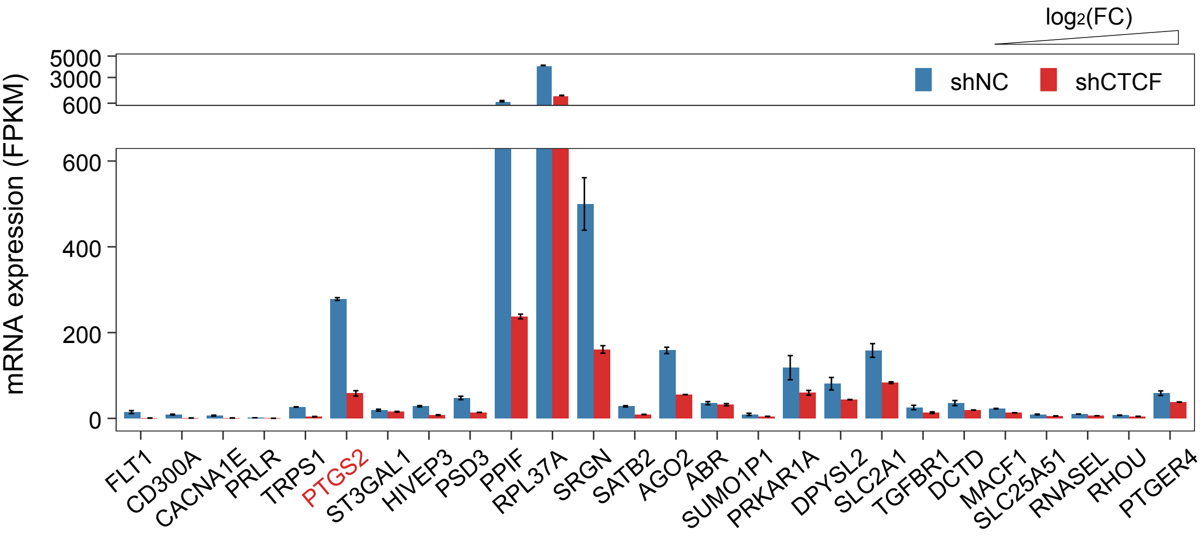

Figure S12

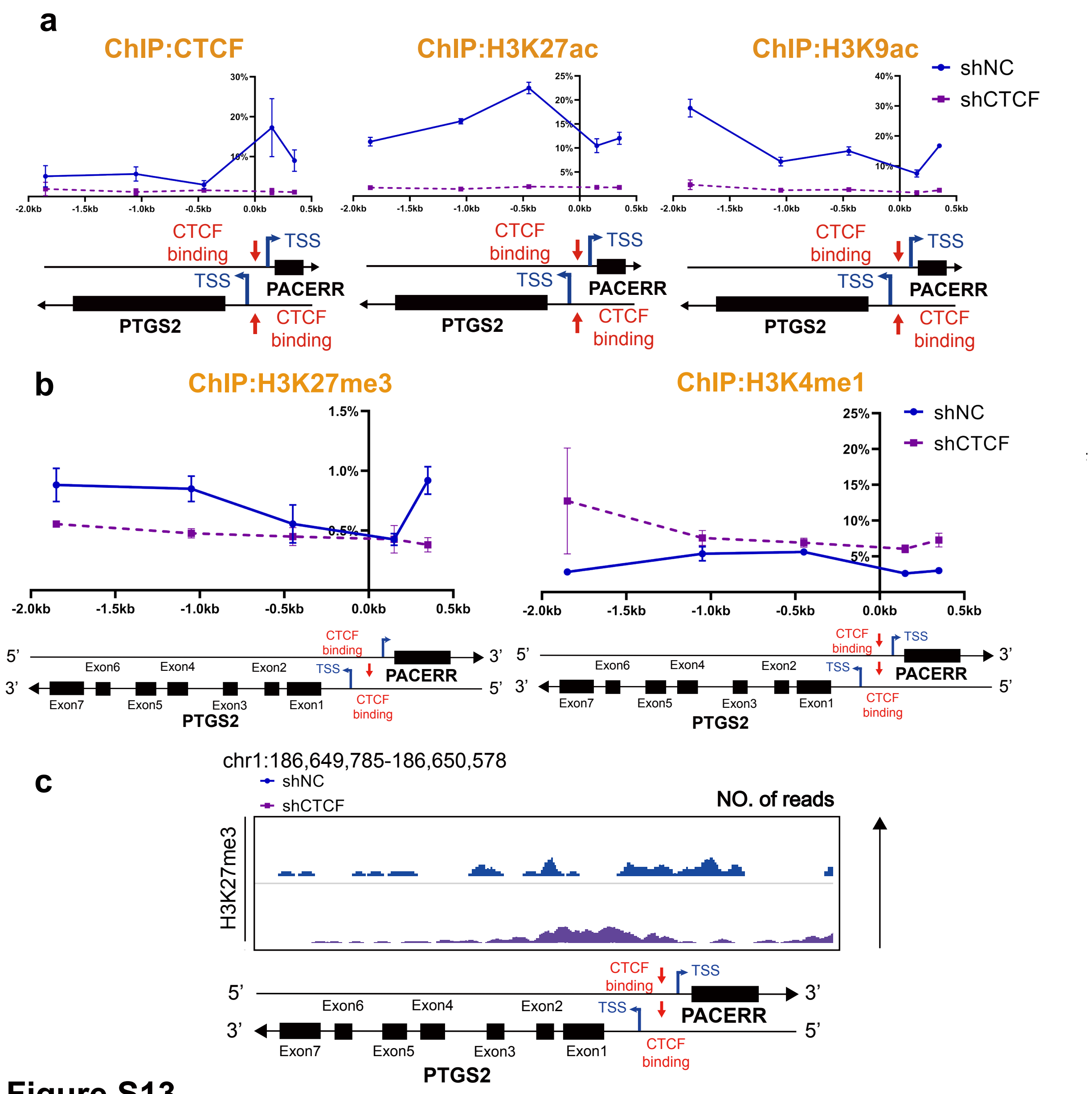
a
ChIP:CTCF
ChIP:H3K27ac
ChIP:H3K9ac
shNC
shCTCF
30%
20%
10%
25%
15%
5%
40%
-2.0kb
-1.5kb
-1.0kb
-0.5kb
0.0kb
0.5kb
CTCF binding
TSS
PACERR
PTGS2
b
ChIP:H3K27me3
ChIP:H3K4me1
1.5%
1.0%
0.5%
5'
3'
Exon6
Exon4
Exon2
Exon7
Exon5
Exon3
Exon1
c
chr1:186,649,785-186,650,578
NO. of reads
H3K27me3

Figure S13

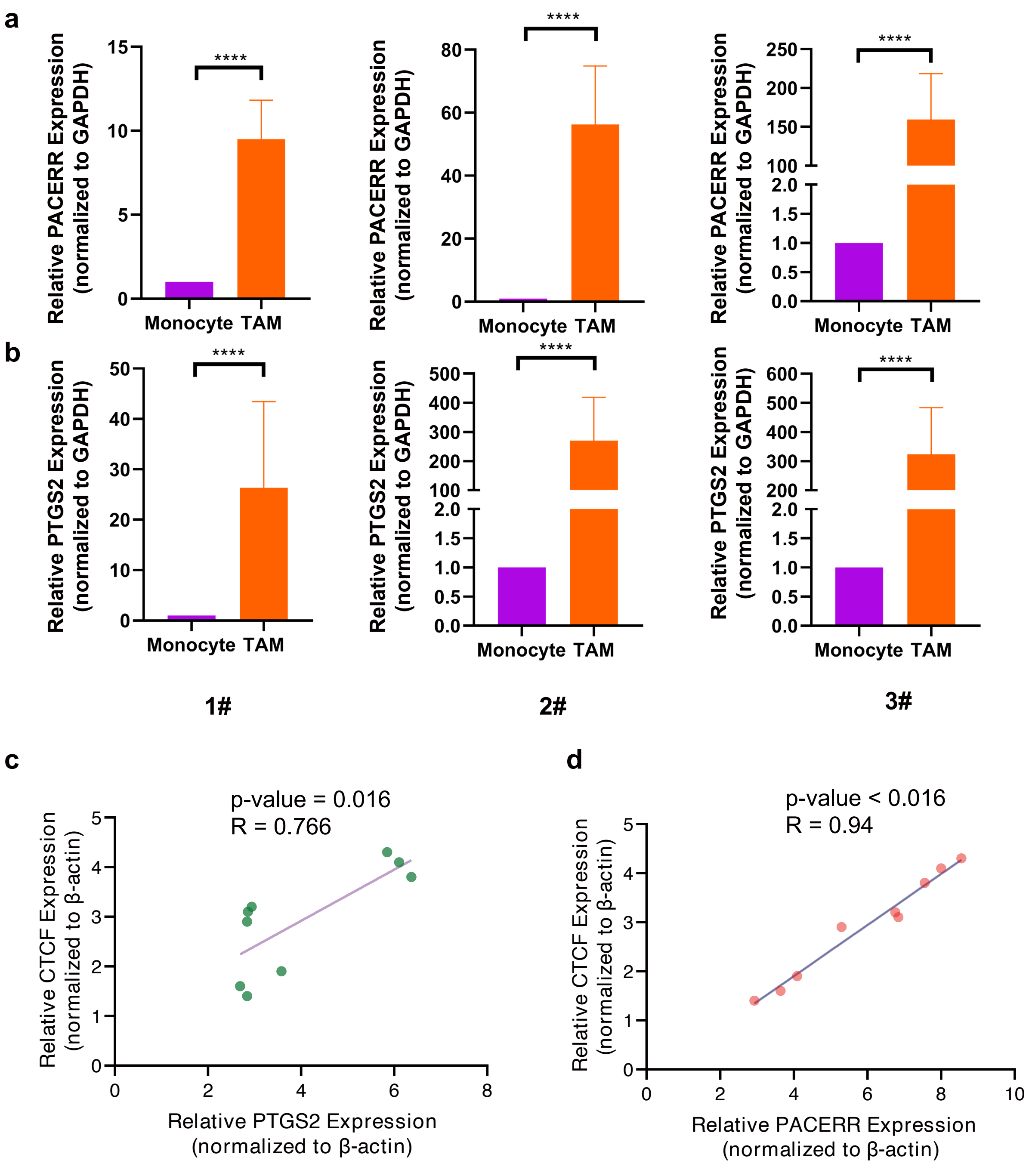
a
b
c
d
Relative PACERR Expression (normalized to GAPDH)
Relative PTGS2 Expression (normalized to GAPDH)
Monocyte TAM
****
1#
2#
3#
p-value = 0.016
R = 0.766
Relative CTCF Expression (normalized to β-actin)
Relative PTGS2 Expression (normalized to β-actin)
p-value < 0.016
R = 0.94
Relative PACERR Expression (normalized to β-actin)

Figure S14

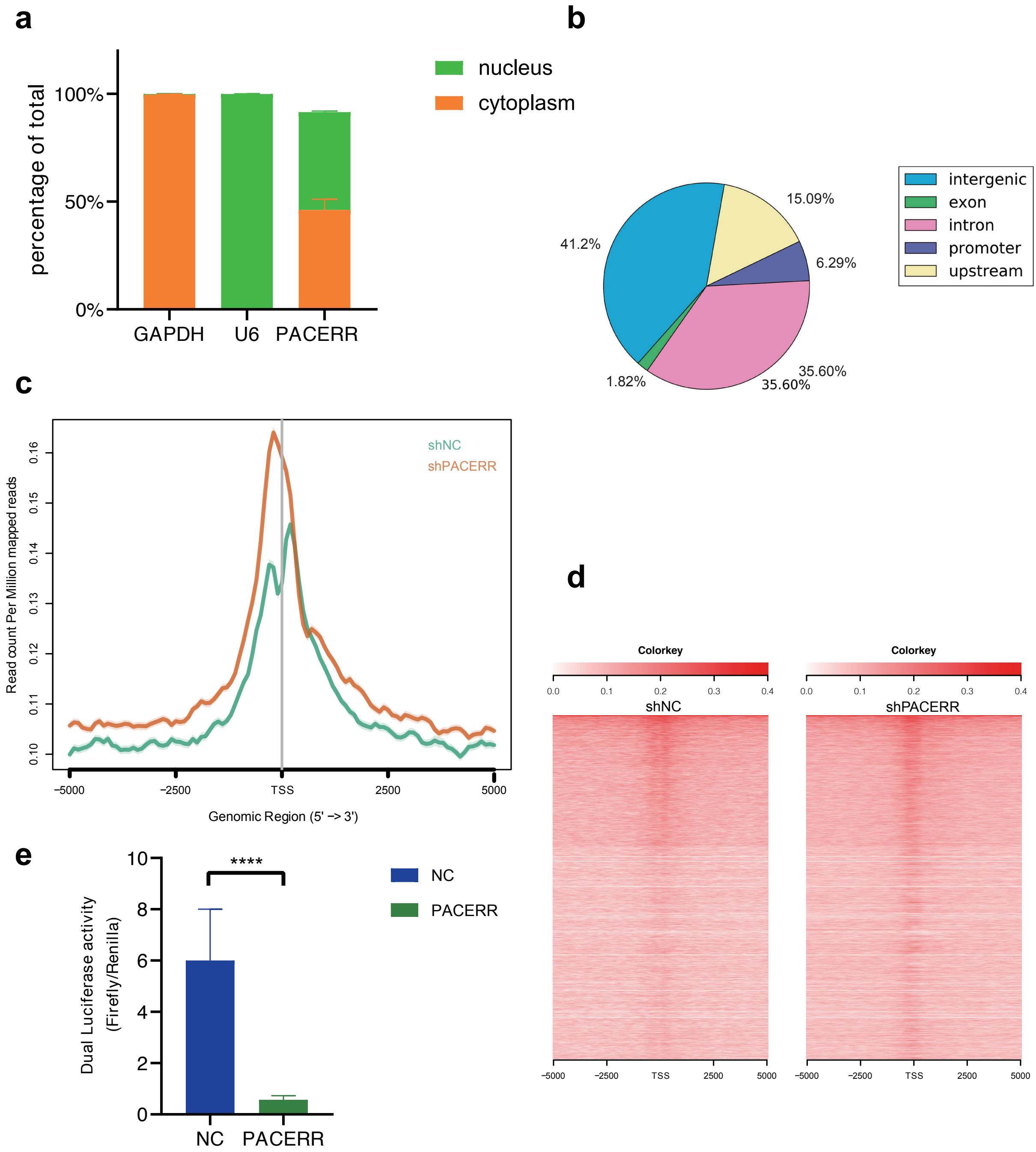

a
percentage of total
100%
50%
0%
GAPDH
U6
PACERR
nucleus
cytoplasm
b
intergenic
exon
intron
promoter
upstream
15.09%
41.2%
6.29%
1.82%
35.60%
35.60%
c
Read count Per Million mapped reads
0.10
0.11
0.12
0.13
0.14
0.15
0.16
shNC
shPACERR
-5000
-2500
TSS
2500
5000
Genomic Region (5' -> 3')
d
Colorkey
0.0
0.1
0.2
0.3
0.4
shNC
shPACERR
-5000
-2500
TSS
2500
5000
e
Dual Luciferase activity
(Firefly/Renilla)
0
2
4
6
8
10
****
NC
PACERR

Figure S15

a

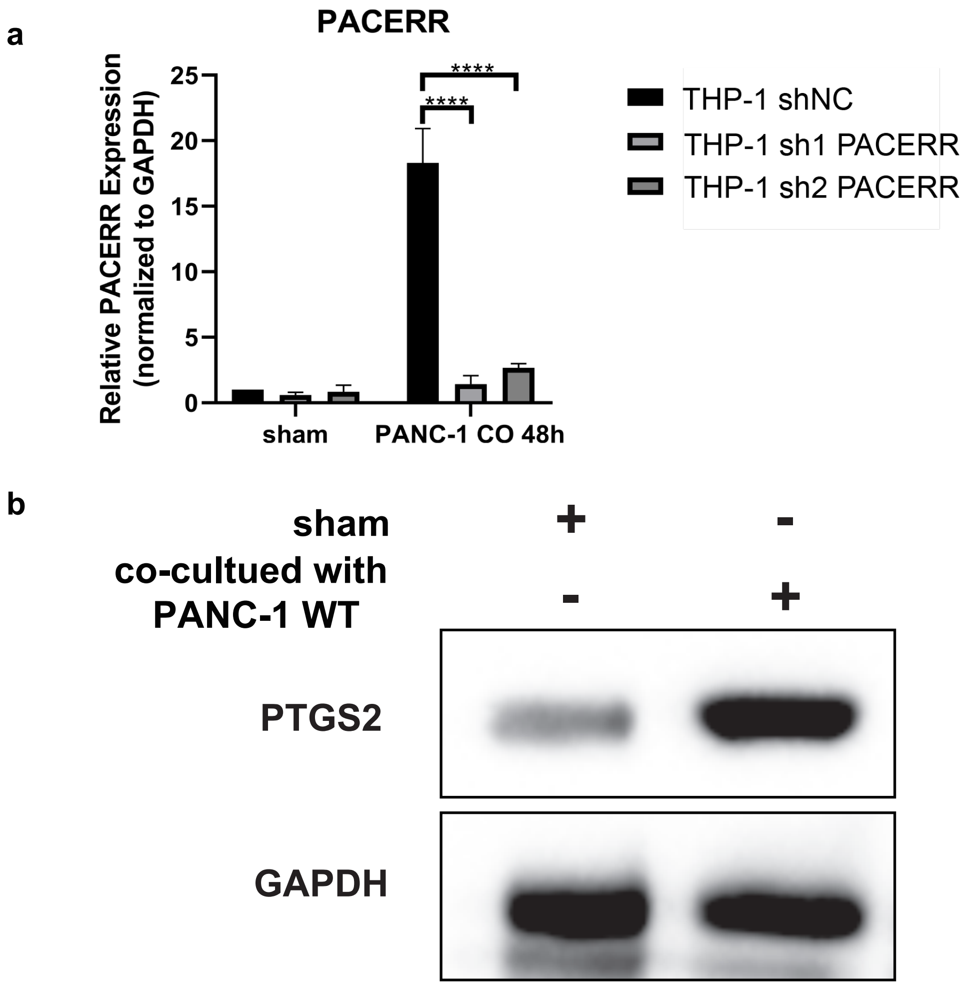
PACERR
Relative PACERR Expression
(normalized to GAPDH)
25
20
15
10
5
0
****
****
sham
PANC-1 CO 48h
THP-1 shNC
THP-1 sh1 PACERR
THP-1 sh2 PACERR
b
sham
co-cultued with
PANC-1 WT
+
-
-
+
PTGS2
GAPDH

Figure S16

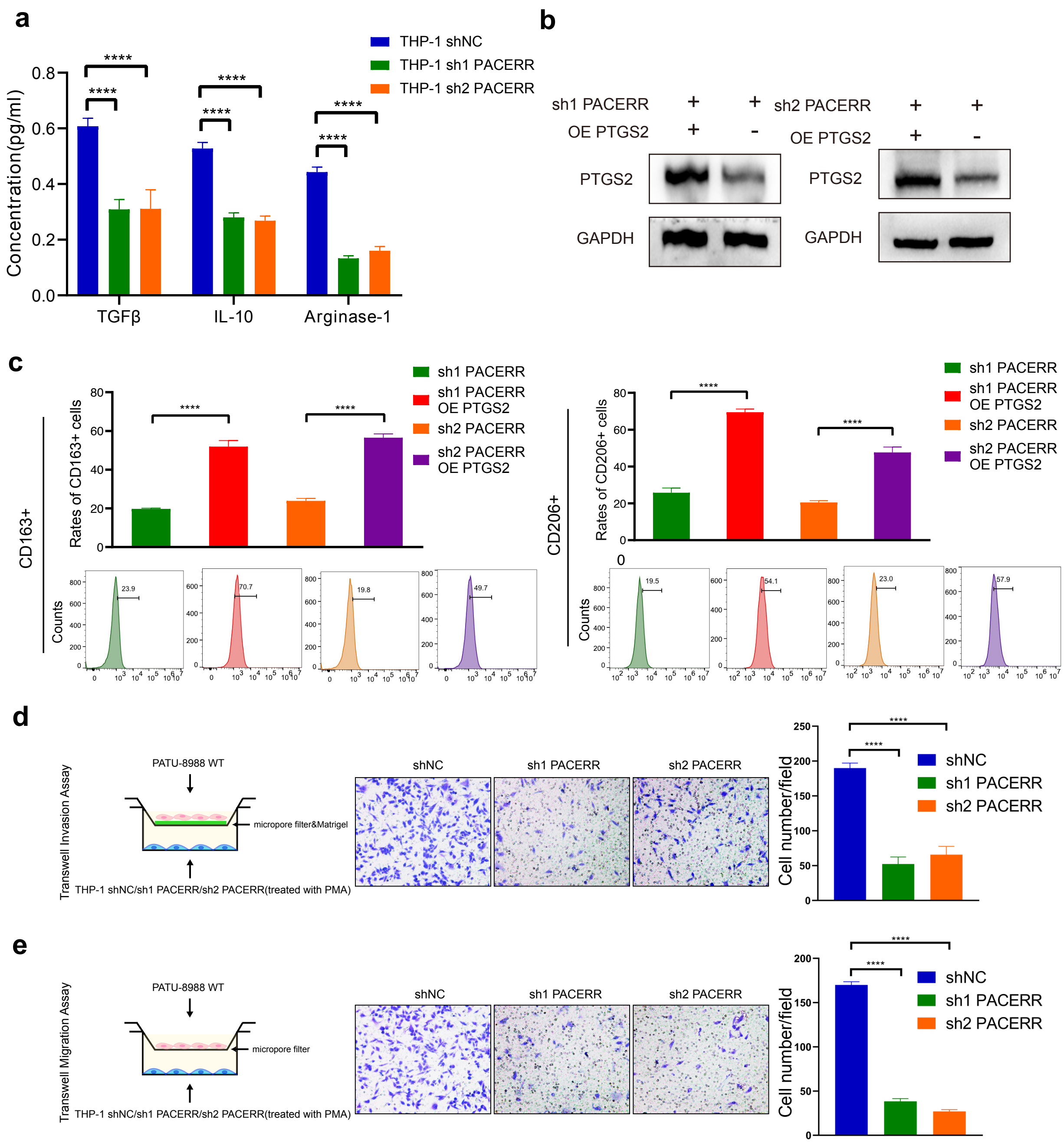

a
THP-1 shNC
THP-1 sh1 PACERR
THP-1 sh2 PACERR
Concentration(pg/ml)
TGFβ
IL-10
Arginase-1
b
sh1 PACERR
OE PTGS2
sh2 PACERR
PTGS2
GAPDH
c
CD163+
Rates of CD163+ cells
CD206+
Rates of CD206+ cells
sh1 PACERR
sh1 PACERR OE PTGS2
sh2 PACERR
sh2 PACERR OE PTGS2
Counts
d
Transwell Invasion Assay
PATU-8988 WT
micropore filter&Matrigel
THP-1 shNC/sh1 PACERR/sh2 PACERR(treated with PMA)
shNC
sh1 PACERR
sh2 PACERR
Cell number/field
e
Transwell Migration Assay
PATU-8988 WT
micropore filter
THP-1 shNC/sh1 PACERR/sh2 PACERR(treated with PMA)
shNC
sh1 PACERR
sh2 PACERR
Cell number/field

Figure S17

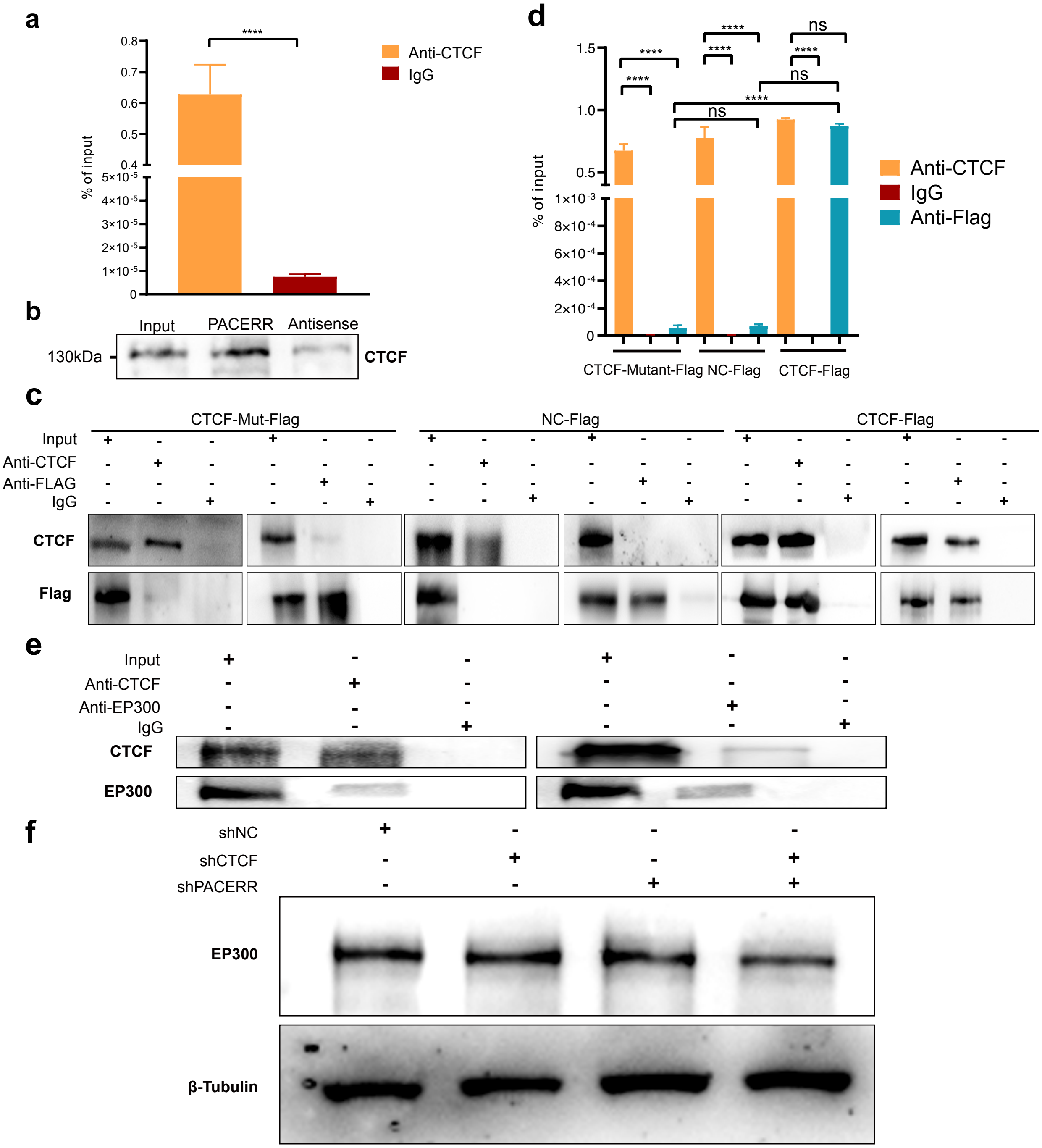

a
****
Anti-CTCF
IgG
% of input
b
Input
PACERR
Antisense
130kDa
CTCF
c
CTCF-Mut-Flag
NC-Flag
CTCF-Flag
Input
Anti-CTCF
Anti-FLAG
IgG
CTCF
Flag
d
ns
****
Anti-CTCF
IgG
Anti-Flag
% of input
CTCF-Mutant-Flag
NC-Flag
CTCF-Flag
e
Input
Anti-CTCF
Anti-EP300
IgG
CTCF
EP300
f
shNC
shCTCF
shPACERR
EP300
β-Tubulin

Figure S18

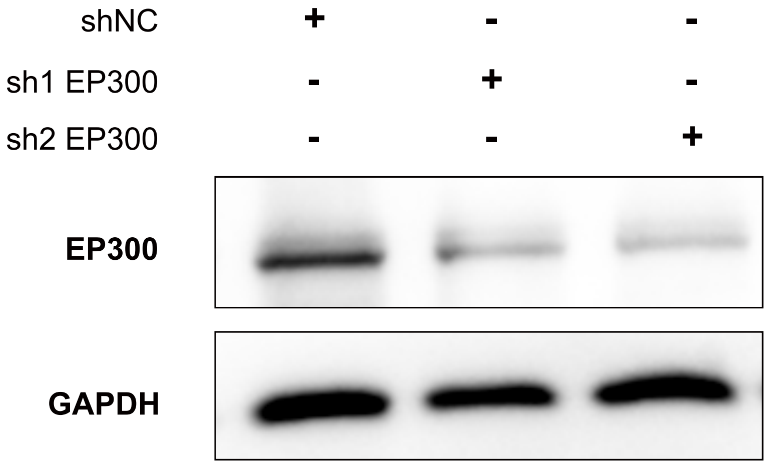

Figure S19

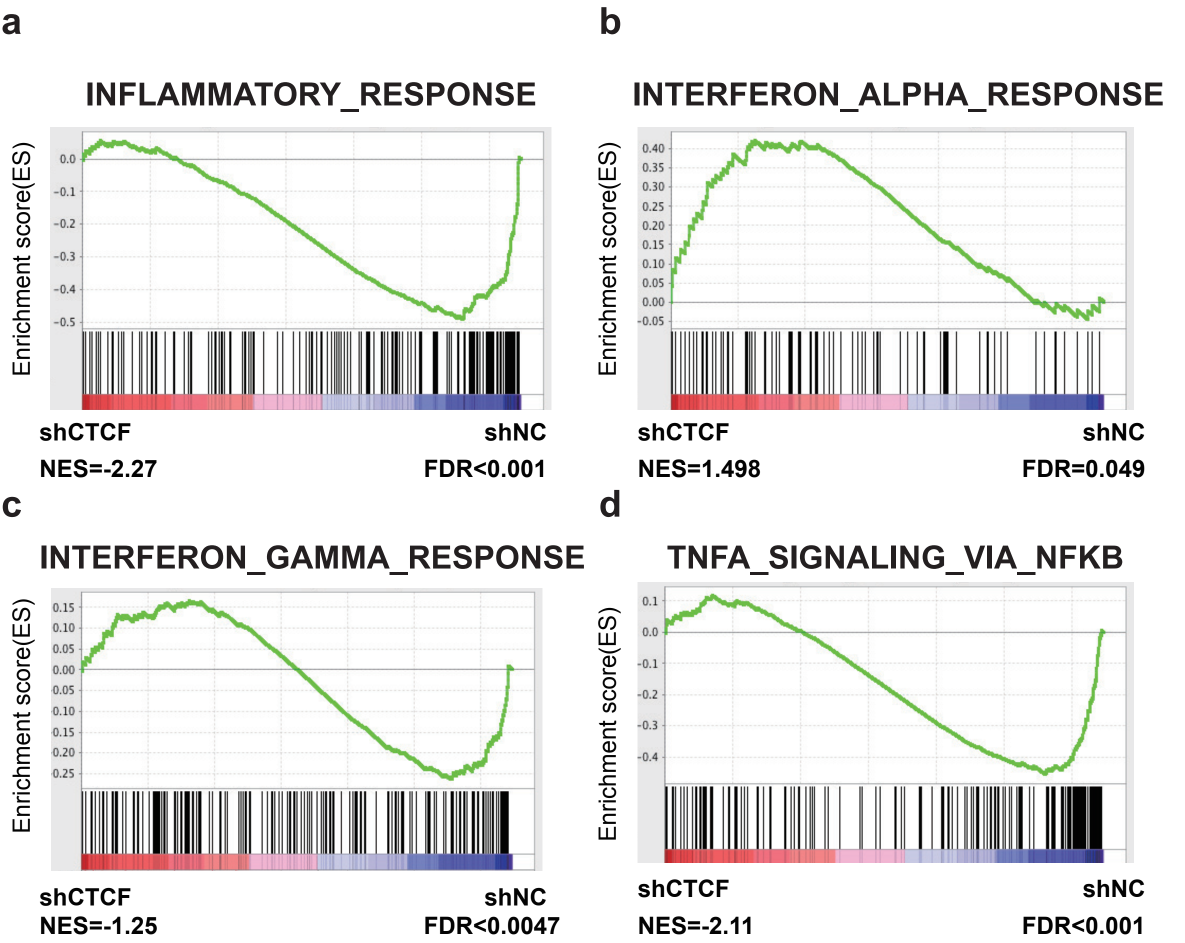
a
INFLAMMATORY_RESPONSE
Enrichment score(ES)
0.0
-0.1
-0.2
-0.3
-0.4
-0.5
shCTCF
shNC
NES=-2.27
FDR<0.001
b
INTERFERON_ALPHA_RESPONSE
Enrichment score(ES)
0.40
0.35
0.30
0.25
0.20
0.15
0.10
0.05
0.00
-0.05
shCTCF
shNC
NES=1.498
FDR=0.049
c
INTERFERON_GAMMA_RESPONSE
Enrichment score(ES)
0.15
0.10
0.05
0.00
shCTCF
shNC
NES=-1.25
FDR<0.0047
d
TNFA_SIGNALING_VIA_NFKB
Enrichment score(ES)
0.1
0.0
-0.1
-0.2
-0.3
-0.4
shCTCF
shNC
NES=-2.11
FDR<0.001

Figure S20

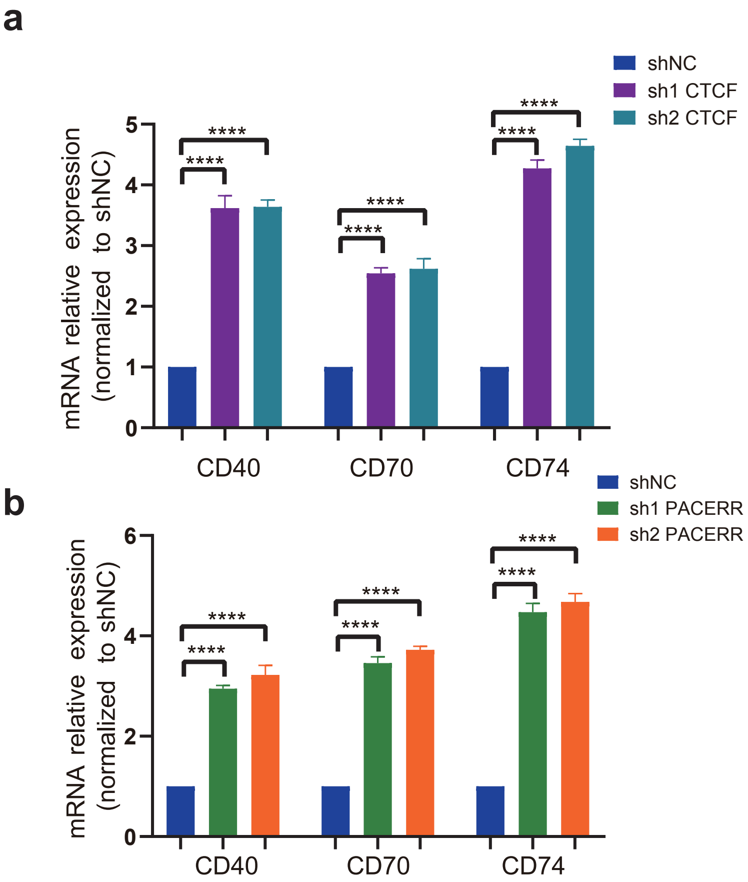
a
shNC
sh1 CTCF
sh2 CTCF
mRNA relative expression (normalized to shNC)
5
4
3
2
1
0
****
****
****
****
****
****
CD40
CD70
CD74
b
shNC
sh1 PACERR
sh2 PACERR
mRNA relative expression (normalized to shNC)
6
4
2
0
****
****
****
****
****
****
CD40
CD70
CD74

Figure S21

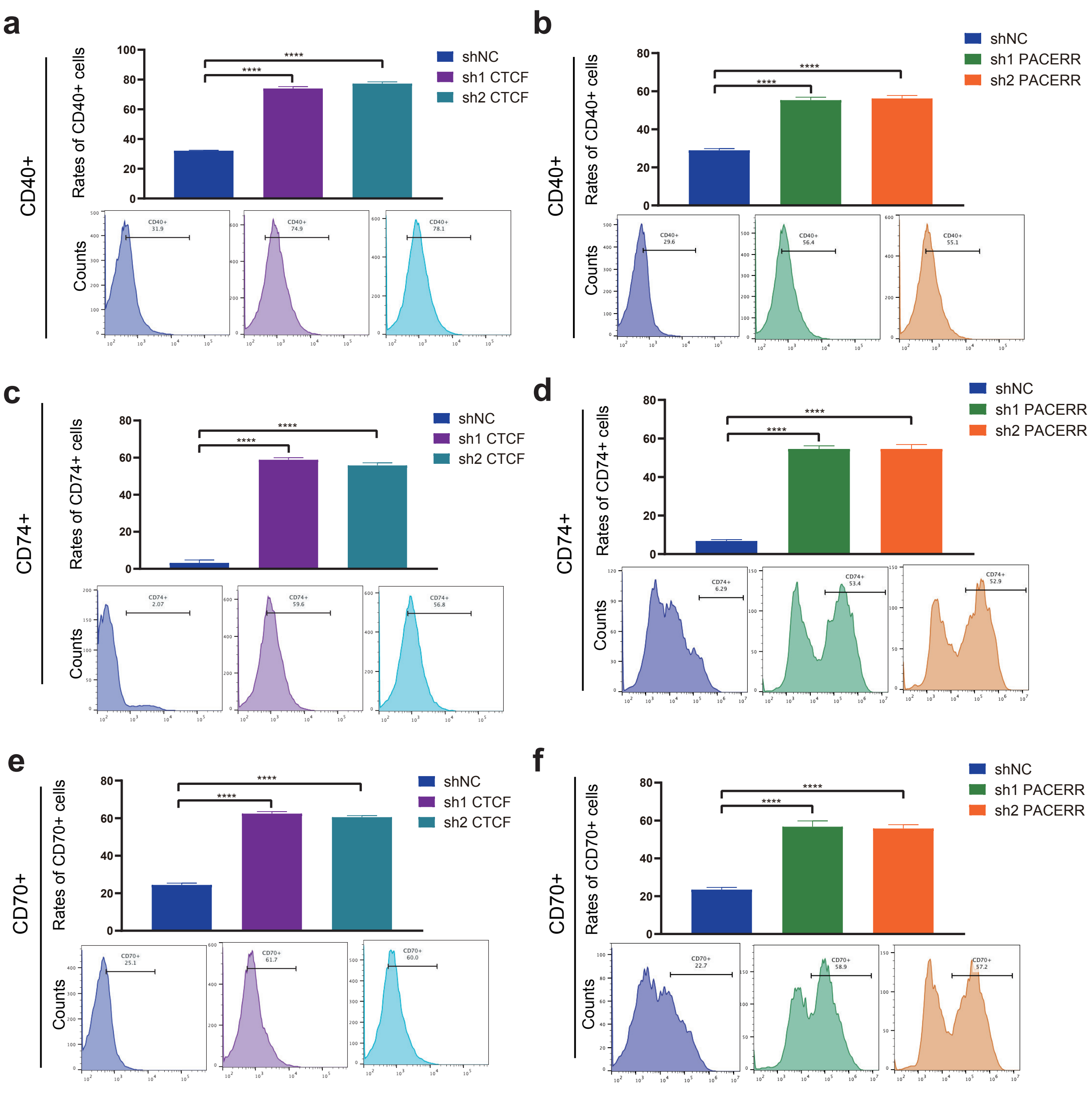
a
CD40+
Rates of CD40+ cells
Counts
shNC
sh1 CTCF
sh2 CTCF
****
CD40+ 31.9
CD40+ 74.9
CD40+ 78.1
b
CD40+
Rates of CD40+ cells
Counts
shNC
sh1 PACERR
sh2 PACERR
****
CD40+ 29.6
CD40+ 56.4
CD40+ 55.1
c
CD74+
Rates of CD74+ cells
Counts
shNC
sh1 CTCF
sh2 CTCF
****
CD74+ 2.07
CD74+ 59.6
CD74+ 56.8
d
CD74+
Rates of CD74+ cells
Counts
shNC
sh1 PACERR
sh2 PACERR
****
CD74+ 6.29
CD74+ 53.4
CD74+ 52.9
e
CD70+
Rates of CD70+ cells
Counts
shNC
sh1 CTCF
sh2 CTCF
****
CD70+ 25.1
CD70+ 61.7
CD70+ 60.0
f
CD70+
Rates of CD70+ cells
Counts
shNC
sh1 PACERR
sh2 PACERR
****
CD70+ 22.7
CD70+ 58.9
CD70+ 57.2

Figure S22
