# Supplementary material for: The CTCF/LncRNA‐PACERR complex recruits E1A binding protein p300 to induce pro‐tumour macrophages in pancreatic ductal adenocarcinoma via directly regulating PTGS2 expression
Source: Clin Transl Med. 2022 Feb 20;12(2):e654. doi: 10.1002/ctm2.654 (PMC8858628; doi:10.1002/ctm2.654)
Supplement: Supplementary file 4 — Supporting Information [file CTM2-12-e654-s004.pdf]

# Supplementary Methods

## RNA-seq data analysis

Quality control of RNA sequencing was performed via FastQC (http://www.bioinformatics.babraham.ac.uk/projects/fastqc/). The sequenced reads were aligned to human genome build hg19 using STAR (1). The mRNA expression levels of all genes were quantified by fragments per kilobase million reads (FPKM) based on RefSeq gene annotation using Cuffdiff (2). Differentially expressed genes (DEGs) were identified by a Q value < 0.05 and fold change (FC) > 1.5. The aligned BAM files were converted to bigWig format and normalized by reads per kilobase million (RPKM) with bamCoverage in deepTools 2.0 (3). ggplot2 (4) from the R package and deepTools 2.0 (3) were used for data visualization and normalization.

## ATAC-seq data analysis

The quality of the sequenced ATAC-seq data was evaluated with FastQC (http://www.bioinformatics.babraham.ac.uk/projects/fastqc/). All sequencing data were mapped onto the hg19 human genome assembly using Bowtie2 (5) with parameter -X 2000. Duplicate reads were removed with SAMtools (6), and only nonduplicate reads kept in the BAM format were used for the subsequent analysis. MACS2 (7) was then used to identify regions of ATAC-seq peaks with the parameters --shift -100 --extsize 200. Picard Tools (v.2.2.4, https://broadinstitute.github.io/picard/) and ggplot2 (4) were used to plot the distribution of paired-end sequencing fragment sizes. For visualization, the BAM files were converted to the bigWig format using the bamCoverage script in deepTools 2.0 (3) with RPKM normalization.

The heatmaps and average profiles were also generated using the plotHeatmap and plotProfile functions in deepTools 2.0 (3).

**ChIP-seq data analysis**

Quality control of each sample was accomplished using FastQC (http://www.bioinformatics.babraham.ac.uk/projects/fastqc/). All ChIP-seq data sets were trimmed and aligned to the human genome to build the hg19 genome using Bowtie2 (5). Duplicate reads were removed with SAMtools (6). MACS2 (7) was used to identify regions of ChIP-seq enrichment over the background in an unbiased manner. For histone modification H3K27ac ChIP-seq, we modified the parameters to facilitate the accurate detection of broad peaks with the parameter --broad. For normalization and visualization of the sequencing data, deepTools 2.0 (3) was used to generate bigWig files, heatmaps and average profiles.

**Annotating regions in the genome**

To annotate the location of ATAC-Seq or ChIP-seq peaks in terms of important genomic features, the annotatePeaks.pl script in HOMER (8) was used to assign the peak files in BED format to promoter-TSS (by default defined from -1 kb to +100 bp of TSS), TTS (by default defined from -100 bp to +1 kb of transcription termination site), intron, intergenic, exon, etc., with default parameters.

**GO and KEGG pathway enrichment analysis**

The Gene Ontology (GO) enrichment and Kyoto Encyclopedia of Genes and Genomes (KEGG) pathway analyses were performed using clusterProfiler (9), an R package for the functional

classification and enrichment of gene clusters using hypergeometric distribution. The enriched terms with a P value < 0.05 were considered significant.

**TF motif enrichment and occurrence analysis**

The findMotifsGenome function in HOMER v4.9 (8) was used to analyse the TF motif enrichment of ATAC-seq peaks in differentially accessible regions (DARs), which was used to predict binding sites for a given TF. The annotatePeaks.pl script was used to identify the nearest genes of predicted TF binding sites and TF motif occurrence probability across a genomic window of ± 500 bp surrounding the peak summits within DARs.

**Gene set enrichment analysis (GSEA)**

To identify pathways that were differentially modulated between TAM and MONO samples and in different differentiation stages, GSEA 4.0.3 was performed using the desktop module available from the Broad Institute (http://www.broadinstitute.org/gsea/). GSEA was performed on the ranked transcript lists using 1000 gene set permutations, the collapse of duplicates to a Max probe, and random seeding. The gene sets used included the Hallmark and GO_BP gene sets (MSigDB v7.2).

**RNA pulldown assay**

RNA pulldown assay was performed as described before (10).

**References**

1. Dobin A, Davis CA, Schlesinger F, Drenkow J, Zaleski C, Jha S, et al. STAR: ultrafast universal RNA-seq aligner. Bioinformatics. 2013;29(1):15-21.

2. Trapnell C, Roberts A, Goff L, Pertea G, Kim D, Kelley DR, et al. Differential gene and transcript expression analysis of RNA-seq experiments with TopHat and Cufflinks. Nat Protoc. 2012;7(3):562-78.

3. Ramírez F, Dündar F, Diehl S, Grüning BA, Manke T. deepTools: a flexible platform for exploring deep-sequencing data. Nucleic Acids Res. 2014;42(Web Server issue):W187-91.

4. Ito K, Murphy D. Application of ggplot2 to Pharmacometric Graphics. CPT Pharmacometrics Syst Pharmacol. 2013;2(10):e79.

5. Langmead B, Salzberg SL. Fast gapped-read alignment with Bowtie 2. Nat Methods. 2012;9(4):357-9.

6. Li H, Handsaker B, Wysoker A, Fennell T, Ruan J, Homer N, et al. The Sequence Alignment/Map format and SAMtools. Bioinformatics. 2009;25(16):2078-9.

7. Zhang Y, Liu T, Meyer CA, Eeckhoute J, Johnson DS, Bernstein BE, et al. Model-based analysis of ChIP-Seq (MACS). Genome Biol. 2008;9(9):R137.

8. Heinz S, Benner C, Spann N, Bertolino E, Lin YC, Laslo P, et al. Simple combinations of lineage-determining transcription factors prime cis-regulatory elements required for macrophage and B cell identities. Mol Cell. 2010;38(4):576-89.

9. Yu G, Wang LG, Han Y, He QY. clusterProfiler: an R package for comparing biological themes among gene clusters. Omics. 2012;16(5):284-7.

10. Zhai S, Xu Z, Xie J, Zhang J, Wang X, Peng C, et al. Epigenetic silencing of LncRNA LINC00261 promotes c-myc-mediated aerobic glycolysis by regulating miR-222-3p/HIPK2/ERK axis and sequestering IGF2BP1. Oncogene. 2021;40(2):277-91.
