# Supplementary material for: The CTCF/LncRNA‐PACERR complex recruits E1A binding protein p300 to induce pro‐tumour macrophages in pancreatic ductal adenocarcinoma via directly regulating PTGS2 expression
Source: Clin Transl Med. 2022 Feb 20;12(2):e654. doi: 10.1002/ctm2.654 (PMC8858628; doi:10.1002/ctm2.654)
Supplement: Supplementary file 5 — Supporting Information [file CTM2-12-e654-s001.docx]

**Additional files**

**Additional file1:**

**Supplementary Figure 1** Analysis of chromatin accessibility in TAMs and monocytes isolated from PDAC patients.

**(A)** and **(C)** The purity of CD206^+^ cells by CD206 microbeads sorting. **(B)** The flow cytometry results of positive rates of CD68 in CD206^+^ cells by MACS from PDAC tissues. **(D)** Distribution of ATAC-seq fragment size in each sample. **(E)** Annotation of ATAC-seq peaks to genomic features: exons, intergenic regions, introns, 3’ UTR, 5’UTR, promoters-TSS, TES and noncoding regions. Peak summits located up to 1 kb upstream and 100 bp downstream of the TSS were determined to be promoter-TSS regions. **(F)** The normalized read density indicating the ATAC-seq signal across a genomic window of ± 2 kb surrounding the peak summits.

Abbreviations: TAM: tumor-associated macrophages; MONO: monocytes; TSS: transcription start site.

**Supplementary Figure 2** CTCF expression in paired monocytes, macrophages in paracancer tissue and TAMs from PDAC patients.

**(A)** Genome browser snapshots of mRNA-seq signals for the genomic regions near CTCF in monocytes and TAMs from 2 PDAC patients. Monocytes were isolated from peripheral blood with CD14 positive selection, while TAMs were isolated from tumor tissues with CD206 positive selection. **(B)** qPCR analysis of CTCF mRNA expression of monocytes and TAMs in 3 PDAC patients (patient 1#, patient 2# and patient 3#). Three independent qPCRs on the RNA from the same three patients. Each image is representative of three independent experiments of technical replicates. ^*^P＜0.05; ^**^P＜0.01; ^***^P＜0.001; ^****^P＜0.0001. **(C)** and **(E)** WB analysis of CTCF protein expression of monocytes, macrophages in paracancer tissue and TAMs in 3 PDAC patients. Macrophages in paracancer tissue were isolated from normal tissues with CD11b positive selection. (D) The purity of CD11b^+^ cells by CD11b microbeads sorting. (F) Staining intensity of CTCF, CD206 and DAPI on the immunofluorescence from TMAs of 110 PDAC patients. Pink represents CTCF. Red represents CD206. Blue represents DAPI. (G) Kaplan-Meier survival curve presenting the overall survival of 110 PDAC patients by immunofluorescence, grouped according to the extent of CTCF^+^ TAM infiltration (the percentage of CTCF^+^ TAMs number in total CD206 positive cells number).

**Supplementary Figure 3** Validation of CTCF knockdown in THP-1 cells. **(A)** and **(B)** Western blot analysis of the CTCF expression and the quantification of the bands in THP-1 cells (shNC/sh1 CTCF/sh2 CTCF). **(C)** The lentivirus of CTCF shRNAs did not significantly change cell growth in THP-1 cells by CCK8 assay. **(D)** The expression of EGFP from THP-1 transfected CTCF shRNAs by fluorescence microscope. Scar bar: 100μm.

**Supplementary Figure 4** Knockdown of CTCF hinders the M2 polarization and pro-tumor functions of THP-1-derived TAMs.

**(A)** ELISA analysis of the relative expression of M2 markers (Arginase-1, TGFβ, and IL-10) in the supernatant from THP-1-derived TAMs after CTCF knockdown. THP-1 cells were treated with PMA and co-cultured with PANC-1 cells for two days. **(B)** Invasion capacity of PATU-8988 cells co-cultured with THP-1-derived TAMs (sh-NC/ sh-CTCF). **(C)** Migration capacity of PATU-8988 cells co-cultured with THP-1-derived TAMs (sh-NC/ sh-CTCF). **(D)** The protein level of CTCF was downregulated after CTCF interfered in BMDMs without co-cultured with tumor cells. (E) RNA expression of M2 markers (Arginase-1, CD163, TGFβ, CD206 and IL-10) in BMDMs after CTCF interfered. **(F)** and **(G)** Representative images of IHC (CD68 and CD206) of liver metastasis and the number of CD68^+^ and CD206^+^ cells in metastatic foci from liver tissues. **(H)** Representative images of liver metastasis and the number of metastatic cells in PDAC mouse model, in which PANC-1 cells mixed with or without TAMs (THP-1 shNC/sh1 CTCF) were injected into the spleens of BALB/c nude mice. ^*^P＜0.05; ^**^P＜0.01; ^***^P＜0.001; ^****^P＜0.0001. “ns” means no statistically significance. Data is shown as the results from three random visions from the metastasis.

**Supplementary Figure 5** CTCF in BMDMs facilitates the liver metastasis of PDAC cells.

**(A)**-**(C)** Representative images of liver metastasis and the number of CD68^+^ and CD206^+^ cells in metastatic foci from liver tissues in PDAC mouse model, in which Pan02 cells mixed with BMDMs (siNC/siCTCF) were injected into the spleens of C57BL/6 mice. siNC means that BMDMs were interfered empty vector. ^*^P＜0.05; ^**^P＜0.01; ^***^P＜0.001; ^****^P＜0.0001. “ns” means no statistically significance. Data is shown as the results from three random visions from the metastasis.

**Supplementary Figure 6** Analysis of RNA-seq data comparing the expression profiles between control and sh-CTCF THP-1-derived TAMs.

**(A)** Expression levels (FPKM values) across all gene protein-coding transcripts (hg19 reference genome) were used to calculate the Jensen Shannon divergence (JSD). **(B)** The gene expression profiles of sh-NC (red) and sh-CTCF (blue) THP-1-derived TAMs. **(C)** Volcano plot shows the transcript levels of differentially expressed genes (DEGs) between sh-NC and sh-CTCF THP-1-derived TAMs. A total of 2110 significantly upregulated genes are shown as red dots, and 1909 downregulated genes are shown as blue dots. **(D)** Heatmap showing all 4019 differentially expressed genes between sh-NC and sh-CTCF THP-1-derived TAMs. **(E)** Bar plot showing the mRNA gene expression levels of CTCF, PTGS2 and PACERR in sh-NC (red) and sh-CTCF (blue) THP-1-derived TAMs.

**Supplementary Figure 7** Data analysis of CTCF ChIP-seq in sh-NC THP-1-derived TAMs.

**(A)** Distribution of ChIP-seq fragment size for CTCF in each sample. **(B)** The normalized read density indicating the CTCF signal across a genomic window 2 kb upstream of the TSS and 2 kb downstream of the TES in shNC THP-1-derived TAMs. **(C)** Pie chart showing the proportion of CTCF ChIP-seq peaks within the indicated genomic regions in sh-NC THP-1-derived TAMs. **(D)** Top 10 enriched known TF motifs in CTCF ChIP-seq peaks in sh-NC THP-1-derived TAMs, with p values estimated in HOMER v4.9.

**Supplementary Figure 8** Analysis of chromatin accessibility in sh-NC and sh-CTCF THP-1-derived TAMs.

**(A)** Distribution of ATAC-seq fragment size in each sample. **(B)** Pie chart showing the proportion of ATAC-seq peaks within the indicated genomic regions in sh-NC (top) and sh-CTCF (bottom) THP-1-derived TAMs. **(C)** The normalized read density indicating the ATAC-seq signal across a genomic window of ± 2 kb surrounding the peak summits in sh-NC (top) and sh-CTCF (bottom) THP-1-derived TAMs. **(D)** Top 15 enriched known TF motifs in ATAC-seq peaks in sh-NC (top) and sh-CTCF (bottom) THP-1-derived TAMs , with p values estimated from HOMER v4.9.

**Supplementary Figure 9** Analysis of ChIP-seq for H3K27ac in sh-NC and sh-CTCF THP-1-derived TAMs.

**(A)** Distribution of ChIP-seq fragment size for H3K27ac in each sample. **(B)** Pie chart showing the proportion of H3K27ac ChIP-seq peaks within the indicated genomic regions in sh-NC (top) and sh-CTCF (bottom) THP-1-derived TAMs. **(C)** The normalized read density indicating H3K27ac signal across a genomic window of ± 2 kb surrounding the ChIP-seq peak summits in sh-NC (top) and sh-CTCF (bottom) THP-1-derived TAMs . **(D)** Top 10 enriched known TF motifs in H3K27ac ChIP-seq peaks in sh-NC (top) and sh-CTCF (bottom) THP-1-derived TAMs, with p values estimated from HOMER v4.9.

**Supplementary Figure 10** Analysis of ChIP-seq for H3K9ac in sh-NC and sh-CTCF THP-1-derived TAMs.

**(A)** Distribution of ChIP-seq fragment size for H3K9ac in each sample. **(B)** Pie chart showing the proportion of H3K9ac ChIP-seq peaks within the indicated genomic regions in sh-NC (top) and sh-CTCF (bottom) THP-1-derived TAMs. **(C)** The normalized read density indicating H3K9ac signal across a genomic window of ± 2 kb surrounding the ChIP-seq peak summits in sh-NC (top) and sh-CTCF (bottom) THP-1-derived TAMs. **(D)** Top 10 enriched known TF motifs in H3K9ac ChIP-seq peaks in sh-NC (top) and sh-CTCF (bottom) THP-1-derived TAMs, with p values estimated from HOMER v4.9.

**Supplementary Figure 11** Analysis of ChIP-seq for H3K27me3 in sh-NC and sh-CTCF THP-1-derived TAMs.

**(A)** Distribution of ChIP-seq fragment size for H3K27me3 in each sample. **(B)** Pie chart showing the proportion of H3K27me3 ChIP-seq peaks within the indicated genomic regions in sh-NC (top) and sh-CTCF (bottom) THP-1-derived TAMs. **(C)** The normalized read density indicating the H3K27me3 signal across a genomic window 5 kb upstream of the TSS and 5 kb downstream of the TES in sh-NC (top) and sh-CTCF (bottom) THP-1-derived TAMs.

**Supplementary Figure 12** Integrative analysis of RNA-seq, ATAC-seq, and ChIP-seq reveals potential downstream targets of CTCF in THP-1-derived TAMs.

**(A)** The number of downregulated differentially expressed genes in sh-CTCF THP-1-derived TAMs, and the number of genes close to differential open sites, CTCF binding sites, H3K27ac modified sites and H3K9ac modified sites, comparing between sh-NC and sh-CTCF THP-1-derived TAMs. **(B)** FPKM of DEGs associated with changes in chromatin accessibility and histone modification between sh-NC and sh-CTCF THP-1-derived TAMs.

**Supplementary Figure 13** PTGS2 is downstream target of CTCF

**(A)** Association of CTCF (left), H3K27ac (middle), and H3K9ac (right) with the promoter region of PTGS2 and PACERR in THP-1-derived TAMs (sh-NC/ sh-CTCF) analyzed by ChIP-qPCR. **(B)** Association of H3K27me3 (left), and H3K4me1 (right) with the promoter region of PTGS2 and PACERR in THP-1-derived TAMs (sh-NC/ sh-CTCF) analyzed by ChIP-qPCR. **(C)** Genome browser snapshots of ChIP-seq (H3K27me3) signals for the genomic regions near PTGS2 and PACERR in THP-1-derived TAMs (sh-NC/ sh-CTCF).

**Supplementary Figure 14** PTGS2 and PACERR RNA expression in monocytes and TAMs and the association between CTCF levels and PTGS2 or PACERR levels in TAMs.

**(A)** and **(B)** qPCR analysis of PACERR and PTGS2 RNA expression in monocytes and TAMs from 3 PDAC patients. Image is representative of three independent experiments. ^*^P＜0.05; ^**^P＜0.01; ^***^P＜0.001; ^****^P＜0.0001. **(C)** and **(D)** Linear regression and Spearman correlation analysis of the relative expression of CTCF and PTGS2 **(C)** or PACERR **(D)** in TAMs isolated from PDAC tissues (n=9). Monocytes were isolated from peripheral blood with CD14 positive selection, while TAMs were isolated from tumor tissues with CD206 positive selection.

**Supplementary Figure 15** Analysis of ChIRP-seq for PACERR in shNC and shCTCF THP-1-derived TAMs.

**(A)** The expression levels of PACERR in the cytoplasm and nucleus in THP-1-derived TAMs (THP-1 WT stimulated with PMA and co-cultured with PANC-1). **(B)** Proportions of PACERR binding sites in each region of the genome in THP-1-derived TAMs (sh-NC/ sh-PACERR THP-1 stimulated with PMA and co-cultured with PANC-1). **(C)** Distribution of peak regions around the TSS (±5 kb to the TSS) in THP-1-derived TAMs (sh-NC/ sh-PACERR THP-1 stimulated with PMA and co-cultured with PANC-1). **(D)** Heatmap showing normalized ChIRP-seq signal (RPKM) in THP-1-derived TAMs (sh-NC/ sh-PACERR THP-1 stimulated with PMA and co-cultured with PANC-1) over differential binding regions (DBRs) of PACERR. Signals within 5 kb surrounding the center of DBRs are displayed in descending order. **(E)** Results of dual luciferase reporter assay of LncRNA-PACERR with promoter region of PTGS2. Image is representative of three independent experiments. ^*^P＜0.05; ^**^P＜0.01; ^***^P＜0.001; ^****^P＜0.0001.

**Supplementary Figure 16** PTGS2 is downstream target of PACERR

**(A)** qPCR validation of PACERR knockdown in THP-1-derived TAMs. Image is representative of three independent experiments. **(B)** PTGS2 protein expression in THP-1 sham (THP-1 stimulated with PMA) and THP-1-derived TAMs (THP-1 stimulated with PMA and co-cultured with PANC-1), examined by Western blot. ^*^P＜0.05; ^**^P＜0.01; ^***^P＜0.001; ^****^P＜0.0001.

**Supplementary Figure 17** Knockdown of PACERR hinders the M2 polarization and the pro-tumor functions of THP-1-derived TAMs.

**(A)** ELISA analysis of the relative expression of M2 markers (Arginase-1, TGFβ and IL-10) in supernatant from THP-1-derived TAMs after PACERR knockdown. THP-1 cells were treated with PMA and cocultured with PANC-1 cells for two days. Image is representative of three independent experiments. **(B)** The expression of PTGS2 were increased after PTGS2 overexpression in THP-1-shPACERR-derived TAMs. **(C)** Flow cytometric analysis of the expression of M2 markers (CD163 and CD206) in THP-1-derived TAMs after PACERR knockdown and PTGS2 overexpression. THP-1 cells were treated with PMA and cocultured with PANC-1 cells for two days. Data is shown as the results from three independent experiments. **(D)** Invasion capacity of PATU-8988 cells co-cultured with THP-1-derived TAMs after PACERR knockdown. (**E**) Migration capacity of PATU-8988 cells co-cultured with THP-1-derived TAMs after PACERR knockdown. ^*^P＜0.05; ^**^P＜0.01; ^***^P＜0.001; ^****^P＜0.0001. Image is representative of three independent experiments.

**Supplementary Figure 18** CTCF binds directly to PACERR and recruits EP300 to the promoter region of PTGS2 in a PACERR-dependent manner in THP-1 cells cocultured with PATU-8988 cells.

**(A)** and **(D)** The results of coimmunoprecipitation (Co-IP) in THP-1-derived TAMs.

Rabbit IgG was used as a negative control. **(A)** RNA immunoprecipitation (RIP) was performed using a CTCF-specific antibody. Eluted CTCF-binding RNAs were reverse transcribed, and qPCR was performed with primers specific for PACERR. Normal rabbit IgG (IgG) was used as a negative control. Data is shown as the results from three independent experiments. **(B)** The validation of CTCF proteins pulled down with PACERR in THP-1 cells cocultured with PATU-8988 by RNA pull-down assay. **(C)** CTCF, Flag and IgG antibodies were used to Co-IP assays. The results of the expression of CTCF and Flag after coimmunoprecipitation (Co-IP) in THP-1-derived TAMs transfected NC-Flag plasmid or CTCF-Mut-Flag plasmid or CTCF-Flag plasmid. Normal rabbit IgG was used as a negative control. **(D)** THP-1 cells cocultured with PATU-8988 were infected with negative control-Flag (only encoding Flag) virus or lentiviral virus encoding Flag-tagged CTCF overexpression transcripts (CTCF-Flag) or Flag-tagged CTCF without the predicted RNA binding region (CTCF-Mutant-Flag) and stimulated into TAM cell models before RIP assays. Whole-cell lysates were subjected to immunoprecipitation with the indicated antibodies. Eluted RNAs were reverse transcribed, and qPCR was performed with primers specific for PACERR. Normal rabbit IgG was used as a negative control. (**E**) CTCF antibody and EP300 antibody were used in Co-IP assay. The results of the expression of CTCF and EP300 after coimmunoprecipitation (Co-IP) in THP-1 co-cultured with PATU-8988. Normal rabbit IgG was used as a negative control. **(F)** Protein levels of EP300 in THP-1-derived TAMs with knockdown of CTCF or dual knockdown of CTCF and PACERR.

**Supplementary Figure 19** Western blot analysis of EP300 protein expression in THP-1-derived TAMs after EP300 knockdown.

**Supplementary Figure 20** The related pathways of inflammation were upregulated in THP-1 derived TAMs after CTCF knockdown.

**(A)**-**(D)** The Hallmarks of inflammatory response **(A)**, interferon alpha response **(B)**, interferon gamma response **(C)** and TNFA signaling via NFKB **(D)** were enriched after CTCF knockdown in TAMs by GSEA analysis.

**Supplementary Figure 21** The RNA levels of CD40, CD70 and CD74 were diminished in THP-1 derived TAMs after CTCF or PACERR knockdown.

**(A) and (B)** qPCR analysis of CD40, CD70 and CD74 in THP-1 derived TAMs after CTCF **(A)** or PACERR **(B)** knockdown. ^*^P＜0.05; ^**^P＜0.01; ^***^P＜0.001; ^****^P＜0.0001. Image is representative of three independent experiments.

**Supplementary Figure 22** Decrease of CTCF or PACERR expression in TAMs resulted in significantly increased percentage of non-classical M1 macrophages.

**(A)**-**(F)** Flow cytometric analysis of CD40, CD70 and CD74 in THP-1 derived TAMs after CTCF or PACERR knockdown. ^*^P＜0.05; ^**^P＜0.01; ^***^P＜0.001; ^****^P＜0.0001. Image is representative of three independent experiments.

**Additional file 2:**

**Table S1:** Sequences targeting CTCF, PACERR and EP300.

**Additional file 3:**

**Table S2:** All primer sequences used in the qPCR process.
